# Capillary leak and endothelial permeability in critically ill patients: a current overview

**DOI:** 10.1186/s40635-023-00582-8

**Published:** 2023-12-20

**Authors:** Babak Saravi, Ulrich Goebel, Lars O. Hassenzahl, Christian Jung, Sascha David, Aarne Feldheiser, Matthias Stopfkuchen-Evans, Jakob Wollborn

**Affiliations:** 1 Department of Anesthesiology, Perioperative and Pain Medicine, Brigham and Women’s Hospital, Harvard Medical School, 75 Francis Street, Boston, MA 02115 USA; 2 Department of Orthopedics and Trauma Surgery, Faculty of Medicine, Medical Center, University of Freiburg, University of Freiburg, Freiburg, Germany; 3 Department of Anesthesiology and Critical Care, St. Franziskus-Hospital, Muenster, Germany; 4 Department of Anaesthesiology, Intensive Care Medicine and Pain Therapy, University Hospital Frankfurt, Goethe University, Frankfurt, Germany; 5 Department of Cardiology, Pulmonology and Vascular Medicine, Heinrich-Heine-University, Duesseldorf, Germany; 6 Institute of Intensive Care Medicine, University Hospital Zurich, Zurich, Switzerland; 7 Department of Anesthesiology, Intensive Care Medicine and Pain Therapy, Evang. Kliniken Essen-Mitte, Huyssens-Stiftung/Knappschaft, University of Essen, Essen, Germany

**Keywords:** Capillary leak syndrome, Critical care, Fluid balance, Endothelial permeability, Angiopoietin-2

## Abstract

Capillary leak syndrome (CLS) represents a phenotype of increased fluid extravasation, resulting in intravascular hypovolemia, extravascular edema formation and ultimately hypoperfusion. While endothelial permeability is an evolutionary preserved physiological process needed to sustain life, excessive fluid leak—often caused by systemic inflammation—can have detrimental effects on patients’ outcomes. This article delves into the current understanding of CLS pathophysiology, diagnosis and potential treatments. Systemic inflammation leading to a compromise of endothelial cell interactions through various signaling cues (e.g., the angiopoietin–Tie2 pathway), and shedding of the glycocalyx collectively contribute to the manifestation of CLS. Capillary permeability subsequently leads to the seepage of protein-rich fluid into the interstitial space. Recent insights into the importance of the sub-glycocalyx space and preserving lymphatic flow are highlighted for an in-depth understanding. While no established diagnostic criteria exist and CLS is frequently diagnosed by clinical characteristics only, we highlight more objective serological and (non)-invasive measurements that hint towards a CLS phenotype. While currently available treatment options are limited, we further review understanding of fluid resuscitation and experimental approaches to target endothelial permeability. Despite the improved understanding of CLS pathophysiology, efforts are needed to develop uniform diagnostic criteria, associate clinical consequences to these criteria, and delineate treatment options.

**Graphical Abstract:**

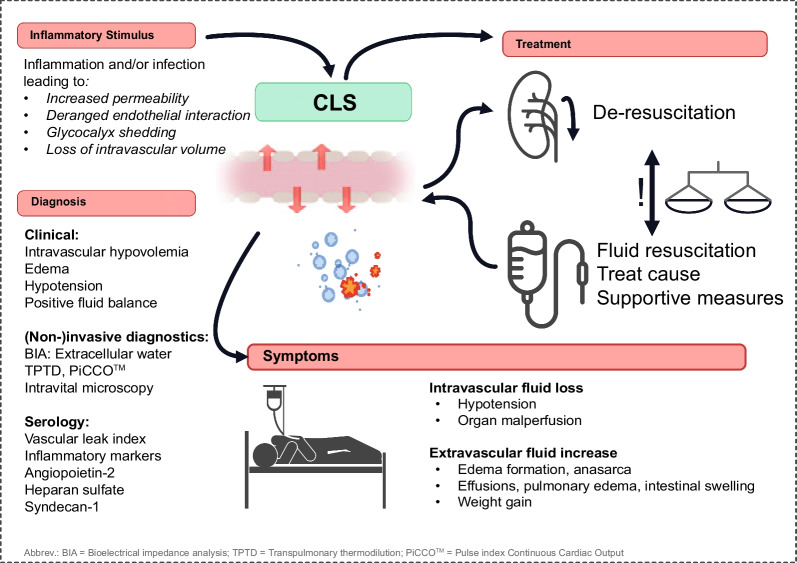

## Introduction

Capillary leak syndrome (CLS) refers to a syndrome of deranged fluid homeostasis, often observed in critically ill [1–3]. In clinical practice, CLS is frequently defined by excessive fluid shift from the intravascular to the extravascular space, resulting in intravascular hypovolemia, extravascular edema formation, and hypoperfusion—necessitating further fluid resuscitation [3].

In health, fluid exchange between intravascular and extravascular spaces is vital for maintaining the body’s homeostasis. However, disturbances in this delicate equilibrium, often driven by systemic inflammation (e.g., sepsis) can lead to the clinical picture of CLS [4–6].

Despite efforts to define CLS, there is no established clinical definition nor accepted diagnostic criteria [1, 3]. Previously, Marx et al. characterized CLS as a fluid extravasation, resulting in edema and hypoperfusion [3]. The authors studied septic shock patients using various methods such as indocyanine green measurements, chromium-51 labeled erythrocytes, and colloid osmotic pressure measurements, aiming to differentiate CLS from other hypo-oncotic conditions and clinical scenarios associated with fluid retention [3]. The definition of CLS proposed by Marx et al. in 2000 emphasized three main aspects: intravascular hypovolemia despite fluid resuscitation, generalized edema, and hemodynamic instability. This pivotal description, while not universally adopted, offers valuable insight into the key features of CLS, aiding in the differentiation of this syndrome from other conditions that share similar clinical manifestations. It underlines the necessity for an accepted definition of this syndrome to develop targeted and effective therapeutic interventions [3].

This article will review the current understanding adding new aspects of CLS in clinical practice, and give an overview about the pathophysiology, clinical presentation, diagnostic and therapeutic options.

## Pathophysiology of CLS and implications

### Triggers and key features

A CLS phenotype can be triggered by numerous disease states as well as certain medications and toxins [7]. Depending on the literature source, terms like “generalized hyperpermeability syndrome”, “endothelial permeability” or “capillary leakage” may be used synonymously for CLS. As an important semantic distinction, the “idiopathic systemic capillary leak syndrome”, also referred as Clarkson’s disease [8], needs to be distinguished from CLS observed in the critically ill with a clear triggering condition. Clarkson’s disease is a rare and potentially fatal condition that is characterized by recurrent episodes of highly acute fluid shifts in otherwise healthy individuals, which can occur in two phases: an initial phase of fluid extravasation associated with syncope, dyspnea, and hypovolemia, followed by a second phase characterized by fluid reabsorption with polyuria and flash pulmonary edema [9]. This condition is rare with limited case reports describing these patients, however the term “systemic capillary leak syndrome” has the potential to cause confusion and impede accurate scientific exchange.

Aside from sepsis triggering CLS [6], other inflammatory states like cardiac surgery using cardiopulmonary bypass [10], anaphylaxis, or major burn injuries can lead to a CLS phenotype. Other rare causes have been described and span from ovarian hyperstimulation syndrome [11, 12], hemophagocytic lymphohistiocytosis [13, 14], viral hemorrhagic fevers [15, 16], autoimmune diseases [17–19], snakebite envenomation [20, 21], and ricin poisoning [22]. Certain drugs, including some interleukins (ILs) [23], monoclonal antibodies and gemcitabine [24], anti-thymocyte globulin may also hold the potential to induce a CLS phenotype [7]. Despite the diverse terminology and triggering factors, the core manifestations remain intravascular hypovolemia, generalized edema, and hemodynamic instability, forming the cornerstone of any definition for this clinical entity.

Due to the heterogenous clinical definition, the relevance of CLS is hard to accurately depict in clinical practice. Good scientific evidence for CLS’ impact on general patient outcomes is lacking. However, its pathophysiological effects on fluid distribution and oxygen transfer can be delineated from a pathophysiological standpoint. Given that endothelial hyperpermeability is a key part of the host’s response to infections, some authors hypothesize that CLS could be a putative target for novel sepsis treatment [25]. A key feature of CLS is represented by capillary permeability, which results in fluid shifts and a decrease in colloid oncotic pressure, often exacerbated by the subsequent fluid resuscitation [26, 27].

Furthermore, an important pathophysiological consideration is the potentially harmful effect on the microcirculation, the network of small vessels crucial for oxygen delivery to the tissues. When fluid leaks out of these vessels into the surrounding tissue, it increases the diffusion distances between capillaries and cells [28]. The systemic implications of CLS underscore the importance of strategies to mitigate the detrimental effects of fluid shifts in key organ systems, maintaining intravascular euvolemia, and ensure adequate oxygenation at the tissue level.

### Inflammatory breakdown of endothelial barrier

Physiologic vascular permeability is a tightly controlled process that is vital to the overall bodily function. However, the extent of permeability varies not only in health and disease, but is also specific to different organ systems and metabolic needs, thereby mirroring each organ's unique biological requirements.

The endothelium is a single layer of cells lining the interior surface of all blood vessels. Its surface area has been estimated to be equivalent to a soccer field [29, 30]. Various endothelial subtypes have been described (e.g., fenestrated, non-fenestrated, sinusoidal), fulfilling specialized and organ-specific functions. It plays an integral role in a diverse array of vascular functions, creating a complex interface between the extra- and intravascular space. Endothelial cells intricately regulate permeability across the endothelium. This is largely due to their ability to form tight, adherens, and gap junctions, the latter of which allow for the exchange of ions, various metabolites, and regulatory factors [31]. Such dynamic functionality marks endothelial activation as a key hallmark for capillary leak [30]. Furthermore, the vascular endothelium acts as a semi-permeable barrier, controlling the exchange of macromolecules and fluids between interstitial fluid and blood. Vascular leakage can occur through two primary pathways [32]: the paracellular and the transcellular pathway [33].

The function of the endothelial barrier varies across different segments of the microvasculature, with permeability increasing from arterioles to venules [34]. There are three types of capillaries: continuous, fenestrated, and discontinuous, which display functional differences specific to the organ [35]. While fenestrated capillaries feature openings with a diameter of 60 nm, their permeability is primarily restricted to water and minor hydrophilic solutes. This limitation is due to the presence of a diaphragm that only allows molecules smaller than 5 nm to pass through [31, 36]. Venules, in contrast, possess endothelial cells with greater permeability characteristics, allowing not just fluids but also solutes and proteins to pass. These cells are particularly responsive to agents that increase permeability [31]. Another factor contributing to the heterogeneity of endothelial permeability across various vascular beds is the extent of coverage by supporting cells, such as pericytes [37]. In the context of inflammation, leukocytes typically exit the bloodstream through venules. This is facilitated by endothelial cells that, when exposed to inflammatory cytokines, present adhesion molecules to which leukocytes can attach. Large veins, however, are less prone to fluid leakage and are not as responsive to agents that increase permeability [38].

**Fig. 1:**
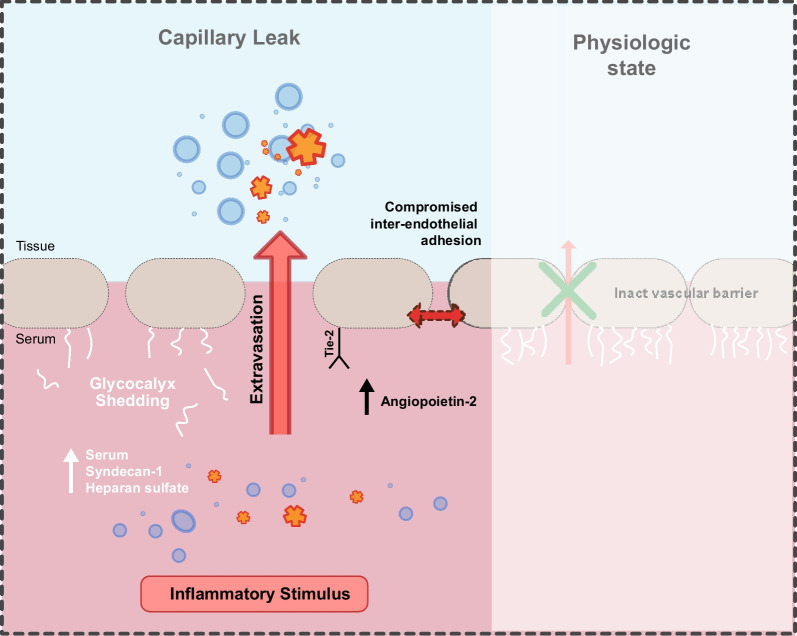
Pathophysiological understanding of selected features of capillary leak including inflammation-induced glycocalyx shedding with increased circulation serum glycocalyx markers (syndecan-1, heparan sulfate), as well as angiopoietin-2/Tie2 signaling leading to compromised inter-endothelial adhesion

In inflammation, the endothelial barrier can be compromised in its integrity (see Fig. 1) [39]. During conditions such as infection (via pathogen-associated molecular patterns, PAMPs) or tissue injury (via damage-associated molecular patterns, DAMPs), endothelial cells undergo a transformation into an activated, proinflammatory state [39]. This activated state is typified by the production and release of various proteins stored in intracellular vesicles known as Weibel–Palade bodies [40]. When endothelial cells are activated, they release proteins such as tissue factor, P-selectin, von Willebrand factor, interleukins, angiopoietin-2 (Ang-2) and many more into the bloodstream [41]. Furthermore, stimulated endothelial cells can produce and distribute proinflammatory cytokines into the bloodstream, which amplifies and exacerbates the inflammatory reaction. In a physiological state, this aims to attract immune cells to localized sites of infection or damage [40]. In case of systemic activation, it may lead to deleterious consequences. Endothelial cells further produce chemoattractants and adhesion molecules, thereby promoting the movement of leukocytes towards inflamed tissues [42, 43]. In a localized inflammatory process, the increase in vascular leakage supports the process of blood cell trafficking and the extravasation of macromolecules to the site. On the local level, this response is beneficial for resolving inflammation and facilitating tissue repair at a given site of an infection [44, 45]. However, when the proinflammatory response escalates to a systemic level, it can lead to a widespread compromise of the endothelial barrier function. This may lead to CLS with relevant fluid shifts, hypotension, intravascular hypovolemia with the need for fluid resuscitation and edema formation. Of note, Kubicki et al. were able to show that CLS in consequence to pediatric cardiac surgery with cardiopulmonary bypass is associated with tissue inflammation as quantified by microdialysis [46]. In case of ongoing hypoperfusion, severe consequences such as organ dysfunction are possible (e.g., acute respiratory distress syndrome, acute kidney injury, etc.) [39, 47, 48]. Furthermore, little is known about the resolution from endothelial dysfunction when inflammatory triggers subside, thus prompting future investigations.

### Pathways in maintaining and compromise of inter-endothelial adhesion

Recent studies have shed light into mechanisms that are involved in destabilizing the endothelial barrier function, prompting the need for innovative therapeutic strategies targeting these pathways to enhance patient outcomes [39]. One identified pathway central to regulation of inter-endothelial adhesions and vascular permeability is the angiopoietin–Tie2 signaling axis [39, 41, 49, 50]. This pathway involves the Tie2 receptor, a second class tyrosine kinase that is almost exclusively expressed by endothelial cells, and its associated ligands, namely angiopoietin 1 (Ang-1) and Ang-2 [39].

Under normal physiological conditions, Tie2 is significantly activated, thereby inhibiting the transcription factor Foxo1, which is responsible for transcribing the Ang-2 gene [51]. However, during inflammation, Ang-2 antagonizes Tie2, disrupting this inhibitory process and permitting Foxo1 to produce more Ang-2 [52]. The activation of Tie2 during vascular quiescence triggers a signaling cascade that strengthens the endothelial cytoskeleton via the inhibition of small GTPases such as RhoA [38, 53]. Nevertheless, this safeguarding effect is negated during inflammation due to the inhibition of Tie2 induced by Ang-2, which results in the production of adhesion molecules and facilitates the migration of immune cells into the inflamed tissue [39, 54]. Another layer of complexity has recently been added to this concept, as it has been observed that not just the activation but also the expression of Tie2 can be severely altered during systemic inflammation [55]. Mechanistically, this could be addressed as a proteolytic shedding process of the Tie2 ectodomain by matrix metalloproteinase 14 [56, 57].

In a resting state, mesenchymal cells, mostly pericytes, secrete Ang-1, which acts as a stimulant for the Tie2 via its phosphorylation, thereby promoting the survival of endothelial cells and maintaining the stability of blood vessels with augmenting its inter-endothelial cellular adhesions [58, 59]. Conversely, Ang-2, stored within the endothelial Weibel–Palade bodies, acts as a context-dependent inhibitor to the Ang1–Tie2 interaction, effectively reducing the activation of the Tie2 receptor [60, 61]. When inflammatory cytokines stimulate the endothelial cells, Ang-2 is released from its storage within the cells, leading to an autocrine deactivation of the Tie2 receptor, a process further intensified by the shedding of the Tie1 ectodomain [62]. It has been observed that Ang-2 levels rise sharply within hours of the onset of sepsis in patients, and these elevated levels have been linked to adverse outcomes and increased mortality rates [63, 64].

It is worth noting that the interplay of these mechanisms result in a reinforcing cycle of inflammation [39]. Besides sepsis, an imbalance in angiopoietin has been associated with negative outcomes in a range of conditions, such as hantavirus, dengue virus, influenza, malaria, and sterile inflammation resulting from extensive surgeries and/or trauma [65–70]. This underscores the potential of the Ang/Tie2 axis as a therapeutic target in various systemic inflammation conditions. Further research into the modulation of this critical pathway may open doors to new therapeutic strategies for systemic inflammation.

Syndecans, comprising Syndecan-1 to -4, are transmembrane proteoglycans essential in endothelial barrier integrity, especially under inflammatory conditions [71]. These proteoglycans interact with various ligands, influencing cell adhesion, angiogenesis, and inflammation [72]. Syndecan-1 and -4 are particularly noteworthy, regulating inflammatory responses in contexts like myocardial injury and sepsis [71, 73]. Syndecan-2 responds to inflammatory stimuli in several cell types, further emphasizing the syndecans' role in inflammation [74, 75]. Syndecan-1 and -4 are pivotal in leukocyte extravasation, facilitating initial rolling and then modulating adhesion and migration to balance inflammation [76–78]. Syndecan-1 also controls leukocyte adhesion to the endothelium, crucial for inflammation regulation [71, 79]. Syndecan-3, while less studied compared to its counterparts, has shown involvement in endothelial function across various vascular beds and influences angiogenesis and vascular permeability [80–83]. In conclusion, current evidence underscores the multifaceted roles of syndecans in endothelial dynamics, particularly emphasizing their significant contribution to inflammation regulation and vascular response under various pathological conditions.

### Glycocalyx shedding

The endothelial glycocalyx (eGC), a coating of sugar molecules on the inner surface of the vascular endothelium, is vital for maintaining vascular stability, fluid homeostasis, and serves as a sophisticated protective shield against inflammation and coagulation [84]. This highly dynamic molecular shield is now viewed as a pivotal player in the pathophysiology of sepsis [85] and has been shown to be compromised in various surgical procedures [10].

In the course of sepsis, the degradation of the glycocalyx takes place through two interrelated "sheddase" mechanisms [85]. These mechanisms pertain to the breakdown of glycosaminoglycans and the cleavage of the core proteoglycans [86–88]. Studies have identified circulating glycosaminoglycans and proteoglycans extracellular domains in sepsis, indicating that these components are released from the glycocalyx, thereby contributing to its thinning and degradation [89–92]. One of the key players in this degradation process is an enzyme known as heparanase-1 [89–92], the only identified mammalian enzyme capable of degrading heparan sulfate polysaccharides into shorter chain oligosaccharides [93]. Heparanase-1 is activated during sepsis, contributing significantly to the degradation of the glycocalyx [85]. The role of heparan sulfate degradation in sepsis, mediated by heparanase-1, has been solidified through numerous preclinical and clinical studies [85, 89–92]. At the same time, septic patients acquire a relevant deficiency of the endogenous heparanase-1 counterpart termed heparanase-2. This dys-equilibrium may represent a novel therapeutic target [94, 95].

Another critical element of the glycocalyx is hyaluronan. Uniquely, hyaluronan is unsulfated and not covalently bound to proteoglycans [96, 97]. Despite its structural differences, hyaluronan plays a crucial role in maintaining the structural stability of the glycocalyx, primarily through its ability to form complexes with proteins and other sulfated glycosaminoglycans [98]. In sepsis, patients have been observed to possess elevated levels of serum hyaluronan, indicating an increase in its degradation [99, 100]. The exact mechanism of hyaluronan degradation in sepsis, however, remains unclear, and this is an active area of research. While there is less understanding regarding the behavior of chondroitin sulfate, dermatan sulfate, and keratan sulfate during sepsis, it is hypothesized that proteoglycans carrying these glycosaminoglycans are expelled from the endothelial glycocalyx during this severe condition [87, 100, 101]. However, the exact mechanism of this shedding process and the identification of the enzymes involved remain as open questions in the field. Further complicating the process, the ectodomains of proteoglycans are also discharged from the endothelial glycocalyx during sepsis [102–104]. This is largely mediated by a group of enzymes known as matrix metalloproteinases (MMPs) and members of the A Disintegrin and Metalloproteinase (ADAMs) family. These enzymes are capable of cleaving proteoglycans from the endothelial glycocalyx and their plasma concentrations are correlated with the severity of sepsis [105–107].

The activation of these sheddase mechanisms is not random. Instead, it is modulated by upstream factors, including proinflammatory cytokines [85]. For instance, sepsis-related activation of the glycosaminoglycans sheddase heparanase-1 is dependent upon endothelial-derived TNF-α [89]. Furthermore, the Ang-2/Tie2 pathway, critical in maintaining endothelial homeostasis, has been shown to regulate glycocalyx sheddases [87, 108–110]. Interestingly, other molecules such as macrophage migratory inhibitor factor, phorbol esters, and tissue inhibitors of matrix metalloproteinases, which are involved in glycocalyx degradation in other diseases, may also be relevant to septic glycocalyx degradation [111–116].

The destructive process of glycocalyx during sepsis has substantial physiological implications. The loss of this protective barrier directly impacts local tissue, but the degradation products themselves can also circulate and affect distant sites in the body [85]. This leads to a system-wide impact that contributes to fluid shifts and the multiple organ dysfunction often seen in septic patients. The extent and the specific mechanisms through which glycocalyx degradation affects the progression and prognosis of sepsis are still being uncovered. This understanding is critical for the development of therapeutic strategies to preserve the eGC, attenuate the inflammatory response, and ultimately improve the outcomes of sepsis.

### Fluid overload and dynamics

The human body contains various fluid compartments, both intravascular and extravascular, which have specific volumes and protein contents. According to indicator dilution studies, a healthy 70 kg adult typically has about 3 L of plasma, containing around 210 g of protein [117]. On the other hand, the same adult will have approximately 12 L of interstitial fluid. This fluid resides in a gel phase and contains 240–360 g of protein. The capillary pressure in this system is higher than the pressure in the interstitial space, which drives the movement of the solvent and its small lipophobic solutes towards the interstitial space [118]. Trans-endothelial fluid shifts are regulated by the vascular barrier in addition to hydrostatic and oncotic forces, as described by the revised Starling equation [119]. In healthy organs, the increased permeability and movement of proteins and plasma fluid are temporary and decrease once the stimulating factor is removed. Edema is traditionally perceived as a consequence of a pressure-driven net outward filtration in the capillary, partially reversed by fluid reabsorption at the venous end by an oncotic pressure gradient [120]. Contrary to traditional perspectives, more recent theories propose that continuous net filtration is the norm in most capillary networks [121]. Apart from an increased pressure gradient, edema can also be caused by hypo-oncotic states, changes in permeability and impaired lympathics. 

The capillary wall includes a glycocalyx layer, which is a complex meshwork of glycosaminoglycans and additional glycoproteins. This layer serves as a filtration barrier, featuring gaps where capillary filtration takes place [121–123]. The movement occurs through regulation of the glycocalyx and the occasional breaks in the inter-endothelial junctions. These breaks constitute less than 0.1% of the total endothelial surface area, allowing a highly regulated fluid exchange process [117]. The glycocalyx layer was previously assumed to have an almost perfect reflection coefficient for proteins, particularly albumin. However, albumin diffusion through capillary pores results in about half of the body's albumin content residing extravascularly, with interstitial oncotic pressure reaching 30–60% of plasma oncotic pressure [124]. The complexity of the interstitial space has been underestimated in the past. It actually consists of a triphasic system that includes freely moving fluids, a gel-like phase rich in large polyanionic glycosaminoglycans molecules, and a collagen framework [117, 124]. Albumin is predominantly absent from this luminal surface, leading to a stronger intravascular oncotic pressure than what direct measurements of interstitial albumin concentration would suggest [125]. As a result, the net filtration process is more influenced by the oncotic pressure beneath the endothelial glycocalyx than by the capillary membrane itself [123].

The clinical consequences of these fluid shifts can be manifold, yet not immediately visible to the clinician. The lungs are especially prone to pulmonary edema due to the unfavorable ratio of endothelium per tissue with the clinical potential to impact gas exchange, and predispose the lungs to further infectious complications [126]. Additionally, the gastrointestinal tract may become edematous, leading to paralytic ileus, an increase in intra-abdominal pressure and subsequent tissue hypoxia, and impaired wound healing [127, 128]. It is noteworthy that the endothelium is highly heterogeneous across different vascular beds; for example, CLS commonly affects various organs but is rarely observed in the brain due to the unique properties of the blood–brain barrier, including a higher pericyte-to-endothelial cell ratio that contributes to its greater impermeability [129].

While CLS is widely acknowledged in the critical care settings, there is a surprising lack of clinical studies exploring its impact on organ dysfunction and mortality [1]. This may stem from the current absence of accepted diagnostic criteria for CLS. However, associated conditions like an inflammatory state and positive fluid balance—circumstances inevitably related to CLS—correlate with higher mortality rates in the ICU [130]. For example, elevated levels of serum cytokines are commonly observed in non-survivors of critically illness, and a positive fluid balance is acknowledged as an independent predictor of outcomes in patients with sepsis [131, 132].

Fluid management can be complex in ICU settings, demanding a thorough understanding of body fluid homeostasis [133]. Fluid overload, which comprises whole body water, i.e., extra- and intravascular fluid, can be detrimental and associated with negative outcomes in patients who are critically ill [134–145]. It has been linked to extended duration of mechanical ventilation [135], increased rate of AKI [136] and renal replacement therapy (RRT) [137], longer ICU stays [135], and increased risk of infectious complications [141]. Furthermore, fluid overload can precipitate intra-abdominal hypertension in ICU patients, regardless of the underlying reason for their admission [142]. In all the aforementioned patient categories, fluid overload is consistently associated with increased mortality rates [134, 137, 138, 140–145]. A systematic review by Messmer et al. in 2020, which encompassed 31 observational and three randomized controlled trials involving a total of 31,076 ICU patients, confirmed a significant correlation between fluid overload and cumulative fluid balance with mortality [146].

While there is a lack of direct evidence on CLS and its impact on patient outcomes, the documented adverse outcomes related to fluid overload strongly underline the importance of further exploring excessive endothelial permeability in the ICU settings. Future research in this area could profoundly influence management strategies and potentially improve outcomes for critically ill patients. Therapeutically, IV fluids may only exert a transient effect on hemodynamics due to their half-life and physiological features to rather liberally cross the vascular barrier [147–149]. It is estimated that less than 5% of infused crystalloid may remain in the vasculature after 1 h [150].

The presence of hypovolemia with peripheral edema represents a counterintuitive scenario that has often baffled physicians. Fluids in the intercellular space can be categorized into two types: unbound fluid and fluid that is part of the gel phase. The gel phase consists of a lattice-like structure made up of collagen and various other fibrous matrix proteins [151]. Fluids are typically free to move between the interstitial space and plasma. Post-filtration, these fluids are channeled back into the circulatory system through the lymphatic network. However, pathological states and certain drugs can disrupt this equilibrium [151]. In inflammatory conditions like sepsis, the fluid's return from the interstitial space towards the plasma may be significantly hindered, leading to the characteristic triad of low blood volume, low albumin levels, and peripheral edema [151, 152]. Even general anesthesia, without the use of mechanical ventilation, has been observed to cause an accumulation of crystalloid fluid, that was previously infused, in a slowly equalizing segment of extravascular spaces [151, 153]. To comprehend the fluid kinetics, it is necessary to integrate data from various fields, including interstitial fluid physiology, lymphatic pathology, and inflammation. It is crucial to understand that the electrolyte composition of the majority crystalloids does not significantly affect kinetics of fluids and, consequently, has limited effects on interstitial fluid pressure [154].

## Diagnostic approach

### Clinical diagnosis

**Fig. 2:**
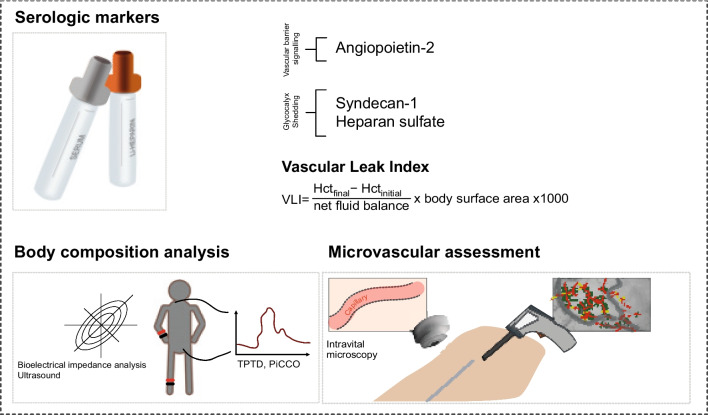
Diagnostic approach to capillary leak syndrome (CLS): both serological markers related to glycocalyx shedding and vascular barrier signaling, as well as the vascular leak index can hint towards a CLS phenotype. More nuanced diagnostic approaches comprise bioelectrical impedance analysis (BIA), transpulmonary thermodilution (TPTD), PiCCO™ (pulse index continuous cardiac output), and intravital microscopy

Diagnosis of CLS is complex (see Fig. 2). So far, no established diagnostic criteria for CLS exist. The need for fluid resuscitation is a critical aspect of CLS diagnosis and management. Clinical hallmarks of CLS may encompass hemodynamic instability, intravascular hypovolemia and generalized edema [7]. Most evident to the clinician at bedside is the systemic pitting edema—but especially effusions in the thoracic and abdominal cavities, non-cardiogenic pulmonary edema, and intestinal swelling can contribute to worse outcomes [155]. While the diagnosis of CLS itself remains a complex endeavor, the assessment of edema, a hallmark feature, presents its own set of challenges. Though various methods for the quantification of peripheral edema exist—ranging from clinical assessments to ultrasound and other advanced imaging modalities—there are no standardized guidelines for the critical care setting [156, 157]. The most common method remains a subjective pitting test, where the severity of edema is graded based on pit depth and skin recovery time [157]. This traditional approach, although quick and widely used, lacks the objectivity and reliability needed for critical assessment.

The management of fluid balance in critically ill patients is a nuanced task. Excessive fluid resuscitation can lead to hypervolemia, ultimately increasing the damage to the glycocalyx and increased vascular permeability [130, 142]. On the other hand, hypovolemia is detrimental for organ perfusion. The diagnosis of CLS can be challenging given the lack of standardized criteria and the varied clinical presentations [1, 3]. Yet, a careful and comprehensive evaluation of patient status, considering the clinical context and use of appropriate diagnostic tools, which will be described below, can assist in identifying the CLS phenotype [1].

### Non-invasive evaluation of extracellular water

Bioelectrical impedance analysis (BIA) offers an approach for non-invasively quantifying the water contents inside and outside cells [158, 159]. This method measures impedance due to the varying electrical conductivity of different biological tissues such as muscle and fat. Given that electrical conductivity correlates with electrolyte and/or water content, BIA can provide a quantitative evaluation of the body's water content, along with fat and muscle mass [159–162].

In a previous study by Marx et al. in critically ill patients, the extracellular water, derived using BIA, correlated well with invasive measurements of extracellular water content [3]. Patients with an elevated extracellular water ratio on the third day in the ICU showed a higher likelihood of postoperative complications and in-hospital mortality [159]. In a recent study conducted on patients undergoing multivisceral debulking surgery, thoracic fluid content, assessed via electrical cardiometry, was found to continuously increase up to the third postoperative day and remained elevated until discharge [163]. This prolonged alteration in fluid status suggests that bioelectrical impedance analysis, including metrics like thoracic fluid content, may offer nuanced insights into volume shifts and their association with postoperative complications. Hence, this non-invasive measurement can be an effective tool for managing volume status, tailoring further therapy, and improving the prognosis for patients in the ICU.

### Serum markers and CLS-scoring system

In their prospective study, Wollborn et al. sought to find common characteristics of CLS in a heterogenous cohort of critically ill patients [1]. They employed a variety of measurement techniques, from non-invasive BIA to serum biomarker analysis, to distinguish patients with CLS from those without [1]. The findings indicated that specific biomarkers previously identified in CLS pathophysiology, particularly Ang-2, showed significantly higher concentrations in CLS patients [1]. Other markers of endothelial integrity, such as the inter-endothelial adherens junction molecule VE-cadherin, and glycocalyx markers like syndecan-1 were elevated in CLS patients as well. In their statistical modellings, Wollborn and colleagues derived a scoring system (“CLS-Score”) which involved seven parameters: ultrasound echogenicity to determine the degree of edema, the Sepsis-related organ failure assessment score (SOFA) score for disease severity, Ang-2, syndecan-1, ICAM-1, lactate, and the proinflammatory cytokine interleukin-6 [1]. By incorporating these components, the score aimed to provide a more objective diagnostic tool for CLS. Many of the identified hallmarks were recently reproduced in a study in cardiac surgery patients [10].

### Vascular Leak Index

As a straightforward yet effective approach to gauge vascular leak in patients suffering from sepsis, Chandra et al. developed the Vascular Leak Index in 2022 [164]. The Vascular Leak Index is calculated using a formula which considers the change in the hematocrit levels at two different timepoints during fluid administration, and the net volume of the administered fluid [164]. In essence, the Vascular Leak Index shows the correlation between the quantity of fluid infused and the change in hematocrit, thereby providing an indication of the amount of fluid that remains in or has escaped from the vascular space. This correlation is normalized to account for differences in each patient's blood volume. The result is then multiplied by 1000 for easier interpretation. By using large ICU databases, the researchers' analysis revealed that higher Vascular Leak Index values are linked to an increased risk of in-hospital death. Furthermore, patients with high Vascular Leak Index values may be at a greater risk of fluid accumulation [164]. A possible limitation is that the Vascular Leak Index cannot differentiate between effects of vascular leak versus concurrent vasodilation or vasoconstriction, especially on the venous side leading to an increase or decrease of venous volume and thereby changing the hematocrit, too [165].

### Invasive assessment of fluid status

Among more invasive diagnostic tools, the use of transpulmonary thermodilution not only presents an approach for hemodynamic monitoring, but also to approximate a patient’s fluid status [166]. Extravascular lung water is defined as the fluid volume outside the pulmonary vasculature, within the interstitial and alveolar spaces [166, 167]. It was previously validated against the reference method of gravimetry in autopsy studies [167–169]. Transpulmonary thermodilution (e.g., with use of the PiCCO™ system) and can be helpful in bedside clinical diagnosis and decision-making. It has been observed that an elevated extravascular lung water is linked to a higher mortality risk in ICU patients [166]. This association held true for both acute respiratory distress syndrome patients and critically ill patients without acute respiratory distress syndrome, suggesting the broad applicability of extravascular lung water as an indicator of disease severity [166]. It is important to clarify that extravascular lung water is a measure of accumulated extravascular water and not a direct indicator of permeability. The Pulmonary Vascular Permeability Index can further distinguish whether the elevated extravascular lung water may be due increased permeability (high Pulmonary Vascular Permeability Index) [170]. It has to be noted that lung-specific pathologies can lead to an increase in extravascular lung water due to localized increase in permeability which may not necessarily represent systemic vascular leak.

### Monitoring endothelial damage and microcirculation

Intravital microscopy utilizing sidestream darkfield or incidental darkfield imaging is gaining popularity for the assessment of the sublingual microcirculation, a non-invasive method that visualizes red blood cells within the microvasculature with light emitted by a light-emitting diode probe, which is then reflected by hemoglobin and detected by a special camera [171]. This technique facilitates the estimation of total vessel density, perfused vessel density, proportion of perfused vessels, and the microvascular flow index, typically through offline computer analysis [172–175].

Moreover, the intravital microscopy imaging of red blood cells serves as a marker of microvascular perfusion, while the measurement of the perfused boundary region provides an indirect marker for endothelial glycocalyx barrier dimensions [125]. Research has shown associations between the perfused boundary region and the presence of red blood cells in the microvascular circulation [176]. This method has further revealed that changes in sublingual microvascular blood flow are prevalent in sepsis patients, with the severity of blood flow abnormality correlating to disease severity [177, 178]. However, these techniques have their limitations, and the low reproducibility of three sublingual microcirculation parameters (vascular density, red blood cell filling, and perfused boundary region) estimated by sidestream darkfield imaging remains a topic of discussion. The recently published DAMIS study showed no benefit on survival by including intravital microscopy in clinical decision-making in patients in shock [172, 175].

Improving technology could further help to visualize the endothelial barrier. For instance, the "GlycoCheck™" camera has been shown as a tool to indirectly evaluate the size of the endothelial glycocalyx [179]. Interestingly, Rovas et al. found that the damage to the endothelial glycocalyx seems to be independent of any microcirculatory disruption as gauged by traditional consensus parameters [179]. This implies that patients can have impaired microcirculation without damage to the endothelial glycocalyx, and vice versa.

## Treatment considerations

### Phases of fluid resuscitation

Importantly, there is no specific treatment for CLS which means tailored therapy needs to focus on nuanced and goal-directed measures to maintain euvolemia and organ perfusion. Fluid administration has to be weighed against potential harm from fluid overload [130, 142], while overzealous fluid resuscitation may further contribute to the degradation of the eGC and subsequently aggravate endothelial injury [108]. While CLS is widely acknowledged in the critical care settings, there is a surprising lack of clinical studies exploring its impact on organ dysfunction and mortality [1]. This may stem from the current absence of accepted diagnostic criteria for CLS. However, associated conditions like an inflammatory state and positive fluid balance—circumstances inevitably related to CLS—correlate with higher mortality rates in the ICU [130]. For example, elevated levels of serum cytokines are commonly observed in non-survivors of critically illness, and a positive fluid balance is acknowledged as an independent predictor of outcomes in patients with sepsis [131, 132].

Fluid management can be complex in ICU settings, demanding a thorough understanding of body fluid homeostasis [133]. Fluid overload, which comprises whole body water, i.e., extra- and intra-vascular fluid, can be detrimental and associated with negative outcomes in patients who are critically ill [134–145]. It has been linked to extended duration of mechanical ventilation [135], increased rate of acute kidney injury [136] and renal replacement therapy [137], longer ICU stays [135], and increased risk of infectious complications [141]. Furthermore, fluid overload can precipitate intra-abdominal hypertension in ICU patients, regardless of the underlying reason for their admission [142]. In all the aforementioned patient categories, fluid overload is consistently associated with increased mortality rates [134, 137, 138, 140–145]. A systematic review by Messmer et al., which encompassed 31 observational and three randomized controlled trials involving a total of 31,076 ICU patients, confirmed a significant correlation between fluid overload and cumulative fluid balance with mortality [146]. Therapeutically, IV fluids may only exert a transient effect on hemodynamics due to their half-life and physiological features to rather liberally cross the vascular barrier [147–149]. It is estimated that less than 5% of infused crystalloid may remain in the vasculature after one hour [150].

**Fig. 3:**
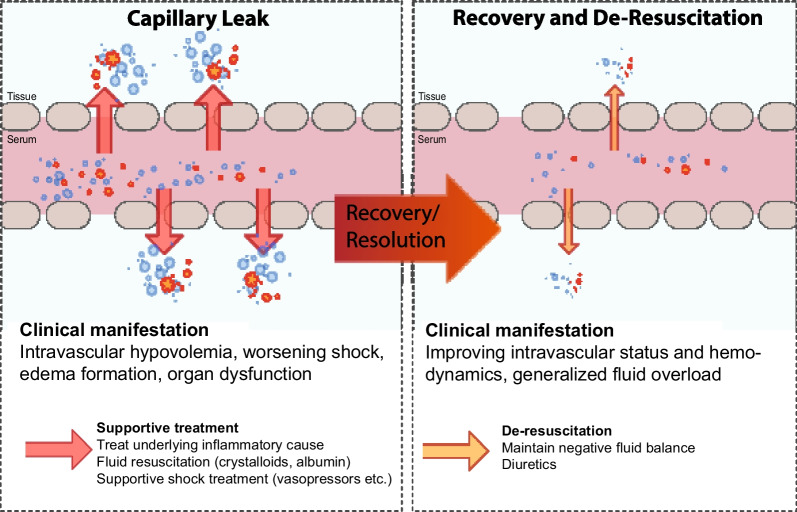
Phases of capillary leak with increased vascular permeability on the left leading to distinct clinical manifestation and necessitating aggressive treatment strategies, while the recovery phase on the right consists of stabilizing and optimizing the fluid status with de-resuscitation

To foster the concept of "fluid stewardship" [180], the ROSE model presents a guide for fluid resuscitation in patients with critical illnesses [130]. It revolves around the four D's—the specific drug (type of fluid), dose (volume), duration, and de-escalation (fluid removal) [130, 180]. These four questions aim to guide clinicians in determining the appropriate timing for initiating and discontinuing fluid therapy, as well as when to begin and cease fluid removal. The four indications refer to the purposes of fluid administration: resuscitation, maintenance, replacement, and nutrition [130]. In the ROSE model, fluid management is conceptualized into four distinct phases: Resuscitation, Optimization, Stabilization, and Evacuation. During the Resuscitation phase, fluids are administered to correct hypovolemia. In the Optimization phase, careful titration of fluids is done to ensure adequate organ perfusion. The Stabilization phase then involves a reduction in fluid administration to prevent fluid overload. Finally, in the Evacuation phase, efforts are made to remove excess fluid and return the patient to normovolemia [130]. A possible parallelism to CLS is that during the optimization phase the fluid administration should be guided to maintain/optimize preload despite the developing CLS. During the stabilization phase the intravascular fluid losses due to CLS should be counterbalanced by a restricted fluid administration, and during the progressive recovery from the CLS permits negative fluid balances during the evacuation phase (see Fig. 3).

### Preservation of the endothelial surface layer (ESL)

In this section, the term 'Endothelial Surface Layer (ESL)' will be used to refer to the intricate structure formed by the endothelial glycocalyx (eGC) along with associated plasma proteins. The eGC serves as a luminal mesh that provides endothelial cells with a framework to bind plasma proteins and soluble glycosaminoglycans [181]. While the eGC itself is considered inactive, it becomes physiologically active once it binds with or is immersed in plasma constituents, thereby forming the ESL. It is worth noting that the specific roles and clinical relevance of the eGC as part of the broader ESL are subjects of ongoing research. The ESL is instrumental in maintaining vascular homeostasis, regulating vascular permeability, and acting as a mechanosensor for hemodynamic shear stresses, in addition to displaying antithrombotic and anti-inflammatory characteristics [182]. Plasma proteins, especially albumin, bind within the glycocalyx and aid in stabilizing this layer [183]. Albumin's function is particularly important as it contributes to plasma colloid osmotic pressure (among other, often unmeasured molecules). Moreover, albumin performs a range of roles—from acting as a free radical scavenger and transporting sphingosine-1-phosphate (which has protective effects on the endothelium), to providing immunomodulatory and anti-inflammatory effects [125].

Experimental studies have highlighted the multifunctional nature of albumin, which includes maintaining ESL integrity, partially restoring compromised vascular permeability, exhibiting anti-oxidative properties and anti-inflammatory properties, improving hemodynamics and microcirculation following endotoxemia or hemorrhagic shock, and acting as an effective plasma volume expander [125, 184–190]. Interestingly, beneficial effects appear to be independent of albumin's oncotic properties. Additional research has shown that the choice of fluid for infusion significantly affects the ESL [125, 191]. For instance, in vivo experiments conducted on anesthetized rats subjected to hemorrhagic shock followed by fluid resuscitation, the use of normal saline failed to restore ESL thickness and plasma levels of syndecan-1 [192]. Conversely, albumin was found to stabilize permeability and leukocyte rolling/adhesion, partially restoring ESL thickness and reducing plasma syndecan-1 to baseline levels [125, 192]. Authors have proposed several mechanisms to elucidate the positive influence of albumin on the endothelium [193]. Primarily, albumin might alleviate sepsis-induced damage to the ESL. As reviewed by Aldecoa et al., albumin, due to its amphoteric properties, has the ability to establish strong bonds with the ESL, while its negative charge aids in maintaining its parietal electrical barrier [125]. In addition, the antioxidant functions of albumin are well-documented [125]. Albumin's free thiol group, carried by a cysteine residue (Cys-34), assists in neutralizing harmful plasma free radicals, which is highly relevant in the septic environment marked by a high oxidative state. Lastly, albumin's capacity to form complexes with heavy metals provides protection against oxidation via the Fenton reaction [193]. Hariri et al. underscore the mounting evidence, both from experimental models and in the context of critically ill patients, that suggests the protective role of albumin on the endothelium during acute injury [193]. Preservation of the ESL using albumin (and fresh frozen plasma) is intriguing, however clinical studies need to confirm these findings. It is anticipated that the ongoing multicenter ARISS trial will further shed light into the effects of albumin on clinical outcomes [194]. It is crucial to note that commercial albumin solutions are often heated to 60 °C for several hours for inactivation of infectious agents [195]. This heat treatment can lead to protein denaturation and alterations in its negative charge [195], raising the question of the comparability of administered albumin with physiologically circulating albumin synthesized by the liver.

Various clinical studies examine the effects of albumin in the clinical context. Zdolsek et al. have shed light on the impact of exogenous albumin administration on fluid dynamics under various clinical conditions [196]. The primary focus of their study was to evaluate the rate at which infused albumin dissipates from the bloodstream, quantified as the half-life (*T*_1/2_), under different clinical scenarios. Their research involved intravenously infusing 3 mL/kg of 20% albumin into a varied population that included healthy volunteers, patients after burns, postoperative patients, and patients who underwent surgery with both minor and significant bleeding. The results showed a consistent T_1/2_ across all groups, except for those who experienced surgery with major bleeding. In the latter case, the infused albumin disappeared faster, indicating a greater loss of albumin in situations of significant hemorrhage. Zdolsek and colleagues further compared the effects of 20% and 5% albumin concentrations on plasma volume expansion [197]. The study was designed in a way that the same mass of albumin was administered under both scenarios. Their findings showed that while both concentrations led to plasma volume expansion, the 5% albumin concentration had a slightly higher rate of volume expansion. However, they found that a third of the 5% albumin solution quickly leaked from the plasma, likely due to the higher colloid osmotic pressure of volunteer plasma than that of the albumin solution. By the 6-h mark, about 42–47% of the administered albumin had leaked from the capillaries, regardless of the concentration used.

Further research by Hahn and colleagues investigated the body fluid shifts when 20% albumin is administered intravenously, with a specific focus on postoperative patients [198]. They found that the infused albumin expanded the plasma volume beyond the volume of the infusion itself by moving non-circulating fluid. However, the same mechanism also increased fluid losses from the system. Despite these dynamics, they observed that the plasma albumin level and plasma volume remained stable for about 2 h post-infusion. Therefore, the effectiveness of albumin as an administered fluid may depend on the specific clinical scenario and the administered concentration.

Microvascular and ESL protection prior to surgeries (i.e., before an anticipated inflammatory insult) presents an interesting area of research, as highlighted by Yanase et al. [199]. In their study, they explored the feasibility, efficacy, and safety of potential protective influence of dexamethasone and albumin on the ESL in patients undergoing abdominal surgery. In this trial, patients were randomly assigned to two groups. One group was given intravenous dexamethasone and 20% albumin at the onset of anesthesia, followed by additional albumin with each subsequent crystalloid administration. The control group, conversely, received only crystalloid fluid without dexamethasone leading to differences in the crystalloid, colloid administration. The outcomes were evaluated based on alterations in plasma syndecan-1 and heparan sulfate levels as markers for eGC damage, and inflammatory markers measured at four perioperative timepoints. Although no significant differences were noted in syndecan-1 levels between the two groups, the group that received the dexamethasone-albumin treatment demonstrated lower heparan sulfate and C-reactive protein levels on the first postoperative day, suggesting a potential protective effect on the glycocalyx. This group also experienced fewer postoperative complications [199]. It remains uncertain if this effect is related to the dexamethasone or albumin administration, or the combination thereof.

It has to be noted that the role of albumin administration in critically ill patients has been studied extensively in the past. The ALBIOS trial conducted by Caironi et al. [200] aimed to evaluate the efficacy of albumin administration in patients with severe sepsis. In this multicenter, open-label trial, 1818 patients with severe sepsis were randomized to receive either a 20% albumin and crystalloid solution or a crystalloid solution alone. The albumin group was targeted to maintain a serum albumin concentration of 30 g per liter or more until discharge from the ICU or 28 days after randomization. During the first 7 days, the albumin group demonstrated a higher mean arterial pressure and a lower net fluid balance compared to the crystalloid group. However, no significant difference was observed in the total daily amount of administered fluid between the two groups. The 28-day and 90-day mortality rates did not show significant differences between the two groups, indicating that albumin replacement in addition to crystalloids did not improve survival rates at these timepoints [200]. These findings do not support the hypothesis that albumin administration has survival benefits in severe sepsis, despite previous studies and experimental evidence for its protective role. However, the ALBIOS trial did confirm some physiological benefits of albumin administration. Patients in the albumin group exhibited superior hemodynamic responses, with a higher mean arterial pressure, lower heart rate, and lower net fluid balance in the first 7 days of treatment [200]. The average cardiovascular SOFA subscore was lower in the albumin group, and the time to suspension of inotropic or vasopressor agents was shorter, suggesting a decreased need for vasopressors [200]. Similar to the ALBIOS trial, the ALBICS trial for albumin use in cardiac surgery did not show a benefit on major adverse events at 90 days [201]. Many unanswered questions remain around the role of albumin administration, e.g., its role in effective de-resuscitation and augmenting loop diuretic effects [202] and the comparability of exogenously administered albumin’s properties compared to that of circulating albumin. Due to these reasons, no final recommendation can be given for the role of albumin administration for CLS treatment.

### Lymphatics in ICU patients

Unlike the cardiovascular system, which ensures bidirectional blood flow, the lymphatic system is specifically designed for unidirectional transit from the extracellular space to the venous system [45]. The lymphatic system plays a pivotal role, actively participating in maintaining tissue fluid equilibrium, aiding in the absorption of lipids from the gastrointestinal tract, and playing an important role in the immune response by transporting antigen-presenting cells and lymphocytes to lymphoid organs [203]. Of note, the lymphatic flow can be increased in health and disease. In the context of critical care, the lymphatic system's potential for increased flow offers interesting avenues for research.

In the critical care setting, physical therapy involving manual lymphatic drainage presents an interesting approach as it has been shown to enhance lymphatic outflow and mobilize fluid [204–206]. Studies have found that manual lymphatic drainage can significantly improve the transportation of various substances within the lymphatic system [204–207]. The findings indicated that manual lymphatic drainage can lead to a modest increase in plasma volume, averaging around 1.5 ± 0.8% [207]. This expansion suggests that lymphatic fluid is being mobilized into the bloodstream. Recent research showed an increase in albumin levels following manual lymphatic drainage [207]. These changes were not solely due to fluid shifts, as albumin concentrations were corrected for changes in plasma volume, and hematocrit remained unaffected by the lymphatic drainage. These observations could imply that the mobilized fluid entering the bloodstream after manual lymphatic drainage therapy possesses a higher albumin content than plasma. The long-term implications of these physiological changes are yet to be fully understood. Nonetheless, the potential role of manual lymphatic drainage in influencing fluid balance and lymphatic outflow could have relevant implications for managing conditions in the ICU.

### Experimental approach for endothelial stabilization

Phosphodiesterase (PDE) inhibitors exhibit a diverse range of pharmacological effects, encompassing properties such as anti-inflammatory, antioxidant, vasodilatory, cardiotonic, and anticancer activities, alongside enhancing memory. This expansive superfamily of PDEs is categorized into 11 distinct groups, differentiated by their structural characteristics, cellular localization, gene expression patterns, protein attributes, and a variety of pharmacological properties, influenced by both internal and external regulatory factors. Particularly, phosphodiesterase-4 inhibitors (PDE4-Is, e.g., rolipram and roflumilast) have been explored as potential treatment options stabilizing endothelial interaction during systemic inflammation and sepsis [208, 209]. The proposed mechanism is thought to involve the control of the cAMP/Rac1-signaling pathway, which is integral to the stability of intercellular junctions [208, 210–212]. The intracellular second messenger cyclic adenosine monophosphate (cAMP) decreases in endothelial cells under inflammatory conditions, associated with the breakdown of endothelial barrier properties in vitro [210]. Experimental studies further suggest that administration of PDE4-Is which increases endothelium-specific cAMP holds the potential to maintain cellular adhesion and endothelial barrier properties during acute inflammation. Schick et al. showed in a rodent model that the application of rolipram or roflumilast effectively attenuated capillary leakage and improved microcirculatory flow by preventing the inflammation-induced loss of endothelial cAMP [208]. Wollborn et al. further confirmed the effects of PDE4-Is in extracorporeal circulation-induced capillary leak [209]. Various other pathways remain under investigation to evaluate means to stabilize vascular endothelium [39].

In addition to PDE4-Is, other PDE-Is also show potential in endothelial stabilization. The PDE1 family, known for its vasodilatory effects and reduced activity in platelet aggregation, may influence endothelial stability by modifying vascular tone and cellular cAMP levels, crucial factors in maintaining endothelial barrier integrity [213–215]. Experimental studies suggest that PDE1 inhibitors, by modulating cGMP and cAMP pathways, could potentially reinforce endothelial cell adhesion and barrier properties, similar to the effects observed with PDE4-Is [216–218]. PDE2-Is, through their unique mechanism of cGMP-mediated cAMP regulation, may also contribute to endothelial stability. By enhancing intercellular communication and barrier function, they could offer a novel approach to managing endothelial disruption in conditions such as pulmonary hypertension and heart failure [219–221]. Furthermore, PDE3-Is, while primarily recognized for their cardiac effects, could indirectly influence endothelial function. Given their role in modulating intracellular cAMP levels, they might impact endothelial cell junction stability and barrier properties, particularly under stress conditions such as sepsis or systemic inflammation [222–224]. Among the most promising for endothelial stabilization are PDE5-Is like sildenafil and tadalafil. These agents have shown effectiveness in improving hemodynamics and endothelial function in heart failure and pulmonary arterial hypertension [225–227]. Their mechanism, which involves modulating cGMP-dependent signaling, makes them particularly relevant for maintaining endothelial barrier integrity. While primarily associated with visual functions, the role of PDE6 in other cellular processes remains under-investigated in the context of endothelial stabilization [228], PDE7-Is are present in immune cells and cardiac myocytes and might influence endothelial function indirectly through immunomodulatory pathways [229, 230]. Both PDE8 and PDE9 are involved in cAMP and cGMP signaling, respectively. While their direct role in endothelial stabilization is not as prominent, they may offer insights into cardiovascular functions and pathologies [231–233]. PDE10 and PDE11 are primarily explored for neurological and psychiatric disorders, and tumor development. Their role in endothelial stabilization is less defined [234, 235].

Recent insights have highlighted the pivotal role of vasodilators, particularly prostaglandins, in regulating endothelial capillary permeability. Prostaglandins, notably prostaglandin E2, play a significant role in this regard. Activation of the prostaglandin E2 receptor signal, which induces vasodilation, could be targeted to enhance endothelial barrier function and counteract capillary leak syndrome [236]. Experimental strategies might involve modulating these pathways to optimize vascular tone and permeability. Endothelium-derived vasodilators, including NO, prostacyclin, and endothelium-derived hyperpolarizing factors, play a central role in maintaining vascular tone. NO, synthesized by endothelial nitric oxide synthase, is instrumental in regulating vascular tone and endothelial function [237–239]. For example, strategies that enhance endothelial nitric oxide synthase activity or NO bioavailability could effectively stabilize endothelial function. This might include gene therapy to upregulate endothelial nitric oxide synthase expression, pharmacological agents to increase NO production, or novel compounds to mimic NO’s vasodilatory effects [236]. Additionally, addressing endothelial hyperpolarization through endothelium-derived hyperpolarizing factors could offer a novel experimental avenue. This might involve manipulating calcium-activated potassium channels or exploring the roles of gap junctions and epoxyeicosatrienoic acids in endothelial cell signaling [240, 241]. Prostacyclin, generated by cyclooxygenase in endothelial cells, activates adenylate cyclase, leading to vascular smooth muscle relaxation [242]. Its role in vasorelaxation suggests potential therapeutic applications in managing endothelial dysfunction. Modulating prostacyclin levels or mimicking its action through pharmacological agents could be an experimental approach to stabilize endothelial cells and maintain vascular homeostasis [236].

Recently, therapeutic plasma exchange has been used in clinical trials to modulate the injurious endothelial activation. The rationale behind this combines two aspects in one procedure: the removal of injurious circulating factors (e.g., Ang-2, heparanase-1) and the replacement of protective factors that have been consumed by the disease process (e.g., heparanase-2 or Ang-1) [243]. This concept has been demonstrated both by quantifying these factors before and after and by ex vivo stimulation of endothelial cells with plasma from these patients [95, 244, 245].

## Conclusion

This review elucidates the multifaceted nature of CLS, underscoring the importance of recognizing its diverse triggers, including systemic inflammation and endothelial barrier breakdown. While current diagnostic methods, such as bioelectrical impedance analysis and serum markers, provide insights, their limitations highlight the need for more precise and universally accepted diagnostic criteria. Treatment strategies, primarily focusing on fluid management and endothelial stabilization, have shown potential, yet they lack specificity and efficacy for CLS. Innovative approaches, like the exploitation of the angiopoietin–Tie2 signaling axis, preservation of the endothelial surface layer, and experimental therapies like phosphodiesterase inhibitors, offer promising directions. Future research should aim to develop a consensus on CLS definition, establish reliable diagnostic benchmarks, and explore these novel therapeutic strategies to enhance patient outcomes in critical care settings.

## Take-home message

CLS presents a diagnostic and therapeutic challenge in critical care due to its complex pathophysiology and the absence of standardized diagnostic criteria. According to the authors of this review, prioritizing research to refine diagnostic tools and explore novel treatments, including endothelial stabilization strategies and experimental pharmacological interventions, is crucial for improving patient management and outcomes in CLS.

## Data Availability

Not applicable.

## References

[1] Wollborn J, Hassenzahl LO, Reker D, Staehle HF, Omlor AM, Baar W (2021). Diagnosing capillary leak in critically ill patients: development of an innovative scoring instrument for non-invasive detection. Ann Intensive Care.

[2] Bichon A, Bourenne J, Gainnier M, Carvelli J (2021). Capillary leak syndrome: state of the art in 2021. Rev Med Interne.

[3] Marx G, Vangerow B, Burczyk C, Gratz KF, Maassen N, Meyer MC (2000). Evaluation of noninvasive determinants for capillary leakage syndrome in septic shock patients. Intensiv Care Med.

[4] Kundra P, Goswami S (2019). Endothelial glycocalyx: role in body fluid homeostasis and fluid management. Indian J Anaesth.

[5] Cordemans C, Laet ID, Regenmortel NV, Schoonheydt K, Dits H, Huber W (2012). Fluid management in critically ill patients: the role of extravascular lung water, abdominal hypertension, capillary leak, and fluid balance. Ann Intensive Care.

[6] Lee WL, Slutsky AS (2010). Sepsis and endothelial permeability. N Engl J Med.

[7] Siddall E, K M and Radhakrishnan, J,  (2017). Capillary leak syndrome: etiologies, pathophysiology, and management. Kidney Int.

[8] Xie Z, Ghosh CC, Patel R (2012). Vascular endothelial hyperpermeability induces the clinical symptoms of Clarkson disease (the systemic capillary leak syndrome. Blood.

[9] Druey KM (2010). Narrative review: the systemic capillary leak syndrome. Ann Intern Med.

[10] Wollborn J, Zhang Z, Gaa J (2023). Angiopoietin-2 is associated with capillary leak and predicts complications after cardiac surgery. Ann Intensive Care.

[11] Schenker JG (1993). Prevention and treatment of ovarian hyperstimulation. Hum Reprod.

[12] Whelan JG, Vlahos NF (2000). The ovarian hyperstimulation syndrome. Fertil Steril.

[13] Ramos-Casals M, Pilar B-Z, Lopez-Guillermo A (2014). Adult haemophagocytic syndrome. Lancet.

[14] Aulagnon F, Lapidus N, Canet E (2015). Acute kidney injury in adults with hemophagocytic lymphohistiocytosis. Am J Kidney Dis.

[15] Giles RB, Sheedy JA, Ekman CN (1954). The sequelae of epidemic hemorrhagic fever; with a note on causes of death. Am J Med.

[16] Peters CJ, Simpson GL, Levy H (1999). Spectrum of hantavirus infection: hemorrhagic fever with renal syndrome and hantavirus pulmonary syndrome. Annu Rev Med.

[17] Natterer J, Perez MH, Bernardo S (2012). Capillary leak leading to shock in Kawasaki disease without myocardial dysfunction. Cardiol Young.

[18] Prete M, Urso L, Fatone MC (2016). Antiphospholipids syndrome complicated by a systemic capillary leak-like syndrome treated with steroids and intravenous immunoglobulins: a case report. Medicine (Baltimore).

[19] Guffroy A, Dervieux B, Gravier S, Martinez C, Deibener-Kaminsky J, Hachulla E (2017). Systemic capillary leak syndrome and autoimmune diseases: a case series. Semin Arthritis Rheum Februar.

[20] Chugh KS (1989). Snake-bite-induced acute renal failure in India. Kidney Int.

[21] Suchithra N, Pappachan JM, Sujathan P (2008). Snakebite envenoming in Kerala, South India: clinical profile and factors involved in adverse outcomes. Emerg Med J.

[22] Bradberry SM, Dickers KJ, Rice P (2003). Ricin poisoning. Toxicol Rev.

[23] Rosenberg SA, Lotze MT, Muul LM (1985). Observations on the systemic administration of autologous lymphokine-activated killer cells and recombinant interleukin-2 to patients with metastatic cancer. N Engl J Med.

[24] Winkler U, Jensen M, Manzke O (1999). Cytokine-release syndrome in patients with B-cell chronic lymphocytic leukemia and high lymphocyte counts after treatment with an anti-CD20 monoclonal antibody (rituximab, IDEC-C2B8). Blood.

[25] Goldenberg NM, Steinberg BE, Slutsky AS, Lee WL (2011). Broken barriers: a new take on sepsis pathogenesis. Sci Transl Med.

[26] Duchesne JC, Kaplan LJ, Balogh ZJ, Malbrain ML (2014). Role of permissive hypotension, hypertonic resuscitation and the global increased permeability syndrome in patients with severe hemorrhage: adjuncts to damage control resuscitation to prevent intra-abdominal hypertension. Anaesthesiol Intensiv Ther.

[27] Cotton BA, Guy JS, Morris JA, Abumrad NN (2006). The cellular, metabolic, and systemic consequences of aggressive fluid resuscitation strategies. Shock.

[28] Ince C (2015). Hemodynamic coherence and the rationale for monitoring the microcirculation. Crit Care.

[29] Augustin HG, Kozian DH, Johnson RC (1994). Differentiation of endothelial cells: analysis of the constitutive and activated endothelial cell phenotypes. BioEssays.

[30] Ait-Oufella H, Maury E, Lehoux S, Guidet B, Offenstadt G (2010). The endothelium: physiological functions and role in microcirculatory failure during severe sepsis. Intensive Care Med.

[31] Claesson-Welsh L, Dejana E, McDonald DM (2021). Permeability of the endothelial barrier: identifying and reconciling controversies. Trends Mol Med.

[32] Duan C-Y, Zhang J, Wu H-L (2017). Regulatory mechanisms, prophylaxis and treatment of vascular leakage following severe trauma and shock. Military Med Res.

[33] Mehta D, Malik AB (2006). Signaling mechanisms regulating endothelial permeability. Physiol Rev.

[34] Palade GE, Simionescu M, Simionescu N (1979). Structural aspects of the permeability of the microvascular endothelium. Acta Physiol Scand Suppl.

[35] Simionescu M (1984) Ultrastructure of the microvascular wall: functional correlations. In: Handbook of physiology, Section “Pathophysiology of CLS and implications” “Introduction”:Chap. 3

[36] Bearer EL, Orci L (1985). Endothelial fenestral diaphragms: a quick-freeze, deep-etch study. J Cell Biol.

[37] Aird WC (2007). Phenotypic heterogeneity of the endothelium: I. Structure, function, and mechanisms. Circ Res.

[38] Wettschureck N, Strillic B, Offermanns S (2019). Passing the vascular barrier: endothelial signaling processes controlling extravasation. Physiol Rev.

[39] Hellenthal KEM, Brabenec L, Wagner N-M (2022). Regulation and dysregulation of endothelial permeability during systemic inflammation. Cells.

[40] Raia L, Zafrani L (2022). Endothelial activation and microcirculatory disorders in sepsis. Front Med.

[41] Milam KE, Parikh SM (2015). The angiopoietin-Tie2 signaling axis in the vascular leakage of systemic inflammation. Tissue Barriers.

[42] Vestweber D (2015). How leukocytes cross the vascular endothelium. Nat Rev Immunol.

[43] Ley K, Laudanna C, Cybulsky MI, Nourshargh S (2007). Getting to the site of inflammation: the leukocyte adhesion cascade updated. Nat Rev Immunol.

[44] Hinsbergh VWM, Collen A, Koolwijk P (2006). Role of fibrin matrix in angiogenesis. Ann N Y Acad Sci.

[45] Cueni LN, Detmar M (2008). The lymphatic system in health and disease. Lymphat Res Biol.

[46] Kubicki R, Grohmann J, Siepe M, Benk C, Humburger F, Rensing-Ehl A (2013). Early prediction of capillary leak syndrome in infants after cardiopulmonary bypass. Eur J Cardio-Thorac Surg.

[47] An R, Pang QY, Liu H (2019). Association of intra-operative hypotension with acute kidney injury, myocardial injury and mortality in non-cardiac surgery: a meta-analysis. Int J Clin Pract.

[48] Matthay MA, Zemans RL, Zimmerman GA, Arabi YM, Beitler JR, Mercat A, Herridge M, Randolph AG, Calfee CS (2019). Acute respiratory distress syndrome. Nat Rev Dis Primer.

[49] Dumont DJ, Yamaguchi TP, Conlon RA, Rossant J, Breitman ML (1992). Tek, a novel tyrosine kinase gene located on mouse chromosome 4, is expressed in endothelial cells and their presumptive precursors. Oncogene.

[50] Dumont DJ, Gradwohl G, Fong GH, Puri MC, Gertsenstein M, Auerbach A, Breitman ML (1994). Dominant-negative and targeted null mutations in the endothelial receptor tyrosine kinase, tek, reveal a critical role in vasculogenesis of the embryo. Genes Dev.

[51] Daly C, Wong V, Burova E, Wei Y, Zabski S, Griffiths J, Lai KM, Lin HC, Ioffe E, Yancopoulos GD (2004). Angiopoietin1 modulates endothelial cell function and gene expression via the transcription factor FKHR (FOXO1. Genes Dev.

[52] Fachinger G, Deutsch U, Risau W (1999). Functional interaction of vascular endothelial-protein-tyrosine phosphatase with the angiopoietin receptor tie-2. Oncogene.

[53] Mammoto T, Parikh SM, Mammoto A, Gallagher D, Chan B, Mostoslavsky G, Ingber DE, Sukhatme VP (2007). Angiopoietin-1 requires P190 RhoGAP to protect against vascular leakage in vivo. J Biol Chem.

[54] Ziegler T, Horstotte J, Schwab C (2013). Angiopoietin 2 mediates microvascular and hemodynamic alterations in sepsis. J Clin Invest.

[55] Ghosh CC, David S, Zhang R (2016). Gene control of tyrosine kinase TIE2 and vascular manifestations of infections. Proc Natl Acad Sci USA.

[56] Thamm K, Schrimpf C, Retzlaff J, Idowu TO, Meurs M, Zijlstra JG, Ghosh CC, Zeitvogel J, Werfel TA, Haller H, Parikh SM, David S (2018). Molecular regulation of acute Tie2 suppression in sepsis. Crit Care Med.

[57] Idowu TO, Etzrodt V, Seeliger B (2020). Identification of specific Tie2 cleavage sites and therapeutic modulation in experimental sepsis. Elife.

[58] Koh GY (2013). Orchestral actions of angiopoietin-1 in vascular regeneration. Trends Mol Med.

[59] Leligdowicz A, Melissa R-G, Wright J, Crowley VM, Kain KC (2018). Endothelial activation: the Ang/Tie axis in sepsis. Front Immunol.

[60] Fiedler U, Scharpfenecker M, Koidl S, Hegen A, Grunow V, Schmidt JM, Kriz W, Thurston G, Augustin HG (2004). The Tie-2 ligand angiopoietin-2 is stored in and rapidly released upon stimulation from endothelial cell Weibel–Palade bodies. Blood.

[61] Mandriota SJ, Pepper MS (1998). Regulation of angiopoietin-2 MRNA levels in bovine microvascular endothelial cells by cytokines and hypoxia. Circ Res.

[62] Korhonen EA, Lampinen A, Giri H, Anisimov A, Kim M, Allen B, Fang S, D’Amico G, Sipilä TJ, Lohela M (2016). Tie1 controls angiopoietin function in vascular remodeling and inflammation. J Clin Investig.

[63] David S, Mukerjee A, Ghosh CC, Yano M, Khankin EV, Wenger JB, Karumanchi SA, Shapiro NI, Parikh SM (2012). Angiopoietin-2 may contribute to multiple organ dysfunction and death in sepsis*. Crit Care Med.

[64] Parikh SM (2017). The angiopoietin-Tie2 signaling axis in systemic inflammation. J Am Soc Nephrol.

[65] Gavrilovskaya IN, Gorbunova EE, Mackow NA, Mackow ER (2008). Hantaviruses direct endothelial cell permeability by sensitizing cells to the vascular permeability factor VEGF, while angiopoietin 1 and sphingosine 1-phosphate inhibit Hantavirus directed permeability. J Virol.

[66] Vuong NL, Lam PK, Ming DKY, Duyen HTL, Nguyen NM, Tam DTH, Duong THK, Chau NV, Chanpheaktra N, Lum LCS (2021). Combination of inflammatory and vascular markers in the febrile phase of dengue is associated with more severe outcomes. Elife.

[67] Lovegrove FE, Tangpukdee N, Opoka RO, Lafferty EI, Rajwans N, Hawkes M, Krudsood S, Looareesuwan S, John CC, Liles WC (2009). Serum angiopoietin-1 and -2 levels discriminate cerebral malaria from uncomplicated malaria and predict clinical outcome in African children. PLoS ONE.

[68] Fremont RD, Koyama T, Calfee CS, Wu W, Dossett LA, Bossert FR, Mitchell D, Wickersham N, Bernard GR, Matthay MA (2010). Acute lung injury in patients with traumatic injuries: utility of a panel of biomarkers for diagnosis and pathogenesis. J Trauma Int Infect Crit Care.

[69] Meyer NJ, Li M, Feng R, Bradfield J, Gallop R, Bellamy S, Fuchs BD, Lanken PN, Albelda SM, Rushefski M (2011). ANGPT2 genetic variant is associated with trauma-associated acute lung injury and altered plasma angiopoietin-2 isoform ratio. Am J Respir Crit Care Med.

[70] Heijden M, Nieu Amerongen GP, Hinsbergh VWM, Groeneveld AJ (2010). The interaction of soluble TIE2 with angiopoietins and pulmonary vascular permeability in septic and nonseptic critically ill patients. Shock.

[71] Gopal S (2020). Syndecans in inflammation at a glance. Front Immunol.

[72] Xian X, Gopal S, Couchman JR (2010). Syndecans as receptors and organizers of the extracellular matrix. Cell Tissue Res.

[73] Götte M (2003). Syndecans in inflammation. FASEB J.

[74] Halden Y, Rek A, Atzenhofer W (2004). Interleukin-8 binds to syndecan-2 on human endothelial cells. Biochem J.

[75] Choi S, Chung H, Hong H (2017). Inflammatory hypoxia induces syndecan-2 expression through IL-1b-mediated FOXO3a activation in colonic epithelia. FASEB J.

[76] Götte M, Joussen AM, Klein C (2002). Role of syndecan-1 in leukocyte–endothelial interactions in the ocular vasculature. Invest Ophthalmol Vis Sci.

[77] Voyvodic PL, Min D, Liu R (2014). Loss of syndecan-1 induces a pro-inflammatory phenotype in endothelial cells with a dysregulated response to atheroprotective flow. J Biol Chem.

[78] Echtermeyer F, Streit M, Wilcox-Adelman S (2001). Delayed wound repair and impaired angiogenesis in mice lacking syndecan-4. J Clin Investig.

[79] Hyun Y-M, Lefort CT, Kim M (2009). Leukocyte integrins and their ligand interactions. Immunol Res.

[80] Arokiasamy S, Balderstone MJM, De Rossi G, Whiteford JR (2020). Syndecan-3 in inflammation and angiogenesis. Front Immunol.

[81] Gopal S, Arokiasamy S, Pataki C (2021). Syndecan receptors: pericellular regulators in development and inflammatory disease. Open Biol.

[82] Tinholt M, Stavik B, Louch W (2015). Syndecan-3 and TFPI colocalize on the surface of endothelial-, smooth muscle-, and cancer cells. PLoS ONE.

[83] Vuong TT, Reine TM, Sudworth A (2015). Syndecan-4 is a major syndecan in primary human endothelial cells in vitro, modulated by inflammatory stimuli and involved in wound healing. J Histochem Cytochem.

[84] Jung C, Fuernau G, Muench P (2015). Impairment of the endothelial glycocalyx in cardiogenic shock and its prognostic relevance. Shock.

[85] Sullivan RC, Rockstrom MD, Schmidt EP, Hippensteel JA (2021). Endothelial glycocalyx degradation during sepsis: causes and consequences. Matrix Biol Plus..

[86] Schmidt EP, Li G, Li L, Fu Li, Yang Y, Overdier KH, Douglas IS, Linhardt RJ (2014). The circulating glycosaminoglycan signature of respiratory failure in critically ill adults. J Biol Chem.

[87] Hippensteel JA, Uchimido R, Tyler PD, Burke RC, Han X, Zhang F (2019). Intravenous fluid resuscitation is associated with septic endothelial glycocalyx degradation. Crit Care.

[88] Fisher J, Linder A, Bentzer P (2019). Elevated plasma glypicans are associated with organ failure in patients with infection. Intens Care Med Exp.

[89] Schmidt EP, Yang Y, Janssen WJ, Gandjeva A, Perez MJ, Barthel L, Zemans RL, Bowman JC, Koyanagi DE, Yunt ZX, Smith LP, Cheng SS, Overdier KH, Thompson KR, Geraci MW, Douglas IS, Pearse DB, Tuder RM (2012). The pulmonary endothelial glycocalyx regulates neutrophil adhesion and lung injury during experimental sepsis. Nat Med.

[90] Wang L, Huang X, Kong G, Xu H, Li J, Hao D, Wang T, Han S, Han C, Sun Y, Liu X, Wang X (2016). Ulinastatin attenuates pulmonary endothelial glycocalyx damage and inhibits endothelial heparanase activity in LPS-induced ARDS. Biochem Biophys Res Commun.

[91] Martin L, Santis R, Koczera P, Simons N, Haase H, Heinbockel L, Brandenburg K, Marx G, Schuerholz T, Stover CM (2015). The synthetic antimicrobial peptide 19–2.5 interacts with heparanase and heparan sulfate in murine and human sepsis. PLoS ONE.

[92] Lygizos MI, Yang Y, Altmann CJ, Okamura K, Hernando AA, Perez MJ, Smith LP, Koyanagi DE, Gandjeva A, Bhargava R, Tuder RM, Faubel S, Schmidt EP (2013). Heparanase mediates renal dysfunction during early sepsis in mice. Physiol Rep.

[93] Vreys V, David G (2007). Mammalian heparanase: what is the message?. J Cell Mol Med.

[94] Pape T, Hunkemöller AM, Kümpers P (2021). Targeting the “sweet spot” in septic shock—a perspective on the endothelial glycocalyx regulating proteins Heparanase-1 and -2. Matrix Biol Plus.

[95] Stahl K, Hillebrand UC, Kiyan Y (2021). Effects of therapeutic plasma exchange on the endothelial glycocalyx in septic shock. Intensive Care Med Exp.

[96] Jung H (2020). Hyaluronidase: an overview of its properties, applications, and side effects. Arch Plast Surg.

[97] Fraser JRE, Laurent TC, Laurent U (1997). Hyaluronan: its nature, distribution, functions and turnover. J Intern Med.

[98] Broekhuizen LN, Mooij HL, Kastelein JJP, Stroes ESG, Vink H, Nieuwdorp M (2009). Endothelial glycocalyx as potential diagnostic and therapeutic target in cardiovascular disease. Curr Opin Lipidol.

[99] Yagmur E, Koch A, Haumann M, Kramann R, Trautwein C, Tacke F (2012). Hyaluronan serum concentrations are elevated in critically ill patients and associated with disease severity. Clin Biochem.

[100] Schmidt EP, Overdier KH, Sun X, Lin L, Liu X, Yang Y, Ammons LA, Hiller TD, Suflita MA, Yu Y, Chen Y, Zhang F, Cothren Burlew C, Edelstein CL, Douglas IS, Linhardt RJ (2016). Urinary glycosaminoglycans predict outcomes in septic shock and acute respiratory distress syndrome. Am J Respir Crit Care Med.

[101] Merline R, Moreth K, Beckmann J, Nastase MV, Zeng-Brouwers J, Tralhao JG, Lemarchand P, Pfeilschifter J, Schaefer RM, Iozzo RV, Schaefer L (2011). Signaling by the matrix proteoglycan decorin controls inflammation and cancer through PDCD4 and MicroRNA-21. Sci Signal.

[102] Bode W, Franz-Xaver G-R, Stockler W (1993). Astacins, serralysins, snake venom and matrix metalloproteinases exhibit identical zinc-binding environments (HEXXHXXGXXH and Met-turn) and topologies and should be grouped into a common family, the ‘metzincins’. FEBS Lett.

[103] Pugin J, Widmer MC, Kossodo S, Liang CM, Preas HL, Suffredini AF (1999). Human neutrophils secrete gelatinase B in vitro and in vivo in response to endotoxin and proinflammatory mediators. Am J Respir Cell Mol Biol.

[104] Nakamura T (1998). Modulation of plasma metalloproteinase-9 concentrations and peripheral blood monocyte mRNA levels in patients with septic shock: effect of fiber-immobilized polymyxin B treatment. Am J Med Sci.

[105] Yang X (2018). A disintegrin and metalloproteinase 15 mediated glycocalyx shedding contributes to vascular leakage during inflammation. Cardiovasc Res.

[106] Dreymueller D, Martin C, Kogel T, Pruessmeyer J, Hess FM, Horiuchi K, Uhlig S, Ludwig A (2012). Lung endothelial ADAM17 regulates the acute inflammatory response to lipopolysaccharide. EMBO Mol Med.

[107] Pruessmeyer J, Martin C, Hess FM, Schwarz N, Schmidt S, Kogel T, Hoettecke N, Schmidt B, Sechi A, Uhlig S, Ludwig A (2010). A disintegrin and metalloproteinase 17 (ADAM17) mediates inflammation induced shedding of syndecan-1 and -4 by lung epithelial cells. J Biol Chem.

[108] Inkinen N, Pettila V, Lakkisto P, Kuitunen A, Jukarainen S, Bendel S, Inkinen O, Ala-Kokko T, Vaara ST (2019). Association of endothelial and glycocalyx injury biomarkers with fluid administration, development of acute kidney injury, and 90-day mortality: data from the FINNAKI observational study. Ann Intensive Care.

[109] Lukasz A, Kumpers P, David S (2012). Role of angiopoietin/tie2 in critical illness: promising biomarker, disease mediator, and therapeutic target?. Sci.

[110] Lukasz A, Hillgruber C, Oberleithner H, Kusche-Vihrog K, Pavenstädt H, Rovas A, Hesse B, Goerge T, Kümpers P (2017). Endothelial glycocalyx breakdown is mediated by angiopoietin-2. Cardiovasc Res.

[111] Nam EJ, Park PW (2012). Shedding of cell membrane-bound proteoglycans. Methods Mol Biol.

[112] Becker BF, Jacob M, Leipert S, Salmon AHJ, Chappell D (2015). Degradation of the endothelial glycocalyx in clinical settings: searching for the Sheddases. Br J Clin Pharmacol.

[113] Lipowsky HH, Lescanic A (2013). The effect of doxycycline on shedding of the glycocalyx due to reactive oxygen species. Microvasc Res.

[114] Manon-Jensen T, Multhaupt HAB, Couchman JR (2013). Mapping of matrix metalloproteinase cleavage sites on syndecan-1 and syndecan-4 ectodomains. FEBS.

[115] Chen H-R, Chao CH, Liu CC, Ho TS, Tsai HP, Perng GC, Lin YS, Wang JR, Yeh TM, Fernandez-Sesma A (2018). Macrophage migration inhibitory factor is critical for dengue NS1-induced endothelial glycocalyx degradation and hyperpermeability. PLoS Pathog.

[116] Fitzgerald ML, Wang Z, Park PW, Murphy G, Bernfield M (2000). Shedding of syndecan-1 and-4 ectodomains is regulated by multiple signaling pathways and mediated by a TIMP-3-sensitive metalloproteinase. J Cell Biol.

[117] Woodcock T (2017). Plasma volume, tissue oedema, and the steady-state Starling principle. BJA Educ.

[118] Wiig H, Rubin K, Reed RK (2003). New and active role of the interstitium in control of interstitial fluid pressure: potential therapeutic consequences. Acta Anaesthesiol Scand Februar.

[119] Michel CC (1997). Starling: the formulation of his hypothesis of microvascular fluid exchange and its significance after 100 years. Exp Physiol.

[120] Little RC, Ginsburg JM (1984). The physiologic basis for clinical edema. Arch Intern Med.

[121] Michel LJ, Michel CC (2010). Microvascular fluid exchange and the revised Starling principle. Cardiovasc Res.

[122] Reed RK, Rubin K (2010). Transcapillary exchange: role and importance of the interstitial fluid pressure and the extracellular matrix. Cardiovasc Res.

[123] Woodcock TE, Woodcock TM (2012). Revised starling equation and the glycocalyx model of transvascular fluid exchange: an improved paradigm for prescribing intravenous fluid therapy. Br J Anaesth.

[124] Bhave GE, Neilson EG (2011). Body fluid dynamics: back to the future. J Am Soc Nephrol.

[125] Aldecoa C, Llau JV, Nuvials X, Artigas A (2020). Role of albumin in the preservation of endothelial glycocalyx integrity and the microcirculation: a review. Ann Intensive Care.

[126] Miranda M, Balarini M, Caixeta D, Bouskela E (2016). Microcirculatory dysfunction in sepsis: pathophysiology, clinical monitoring, and potential therapies. Am J Physiol Heart Circul Physiol.

[127] Attuwaybi B, Kozar RA, Gates KS, Moore-Olufemi S, Sato N, Weisbrodt NW (2004). Hypertonic saline prevents inflammation, injury, and impaired intestinal transit after gut ischemia/reperfusion by inducing heme oxygenase 1 enzyme. J Trauma.

[128] Balogh Z, Mckinley BA, Cocanour CS, Kozar RA, Valdivia A, Sailors RM (2003). Supranormal trauma resuscitation causes more cases of abdominal compartment syndrome. Arch Surg J.

[129] Galea I (2021). The blood–brain barrier in systemic infection and inflammation. Cell Mol Immunol.

[130] Malbrain MLNG, Malbrain G, Ostermann M (2022). Everything you need to know about deresuscitation. Intensive Care Med.

[131] Dimopoulou I, Orfanos S, Kotanidou A, Livaditi O, Giamarellos-Bourboulis E, Athanasiou C (2008). Plasma pro- and anti-inflammatory cytokine levels and outcome prediction in unselected critically ill patients. Cytokine.

[132] Acheampong A, Vincent JL (2015). A positive fluid balance is an independent prognostic factor in patients with sepsis. Crit Care Lond Engl.

[133] Samoni S, Vigo V, Bonilla Reséndiz LI, Villa G, Rosa S, Nalesso F (2016). Impact of hyperhydration on the mortality risk in critically ill patients admitted in intensive care units: comparison between bioelectrical impedance vector analysis and cumulative fluid balance recording. Crit Care.

[134] Boyd J, Forbes J, Nakada T, Walley K, Russell J (2011). Fluid resuscitation in septic shock: a positive fluid balance and elevated central venous pressure are associated with increased mortality. Crit Care Med.

[135] Wiedemann HP, Wheeler AP, National Heart, Lung, and Blood Institute Acute Respiratory Distress Syndrome (ARDS) Clinical Trials Network (2006). Comparison of two fluid-management strategies in acute lung injury. N Engl J Med.

[136] Liu KD, Thompson BT, Ancukiewicz M, Steingrub JS, Douglas IS, Matthay MA (2011). Acute kidney injury in patients with acute lung injury: impact of fluid accumulation on classification of acute kidney injury and associated outcomes. Crit Care Med.

[137] Payen D, Pont AC, Sakr Y, Spies C, Reinhart K, Vincent JL (2008). A positive fluid balance is associated with a worse outcome in patients with acute renal failure. Crit Care.

[138] Bouchard J, Soroko SB, Chertow GM, Himmelfarb J, Ikizler TA, Paganini EP, Mehta RL (2009). Fluid accumulation, survival and recovery of kidney function in critically ill patients with acute kidney injury. Kidney Int.

[139] Heung M, Wolfgram DF, Kommareddi M, Hu Y, Song PX, Ojo AO (2012). Fluid overload at initiation of renal replacement therapy is associated with lack of renal recovery in patients with acute kidney injury. Nephrol Dial Transplant.

[140] Bellomo R, Cole A, Cole L, Finfer S, Gallagher M, Lee J, Lo S, McArthur C, McGuiness S, Norton R, Myburgh J, Scheinkestel C, Su S (2012). An observational study fluid balance and patient outcomes in the randomized evaluation of normal vs. augmented level of replacement therapy trial. Crit Care Med.

[141] Barmparas G, Liou D, Lee D, Fierro N, Bloom M, Ley E, Salim A, Bukur M (2014). Impact of positive fluid balance on critically ill surgical patients: a prospective observational study. J Crit Care.

[142] Malbrain ML, Marik PE, Witters I, Cordemans C, Kirkpatrick AW, Roberts DJ, Van RN (2014). Fluid overload, de-resuscitation, and outcomes in critically ill or injured patients: a systematic review with suggestions for clinical practice. Anaesthesiol Intensive Ther.

[143] Vincent JL, Sakr Y, Sprung CL, Ranieri VM, Reinhart K, Gerlach H, Moreno R, Carlet J, Le Gall JR, Payen D (2006). Sepsis in European intensive care units: results of the SOAP study. Crit Care Med.

[144] Vaara ST, Korhonen AM, Kaukonen KM, Nisula S, Inkinen O, Hoppu S, Laurila JJ, Mildh L, Reinikainen M, Lund V, Parviainen I, Pettila V, Finnaki SG (2012). Fluid overload is associated with an increased risk for 90-day mortality in critically ill patients with renal replacement therapy: data from the prospective FINNAKI study. Crit Care.

[145] Wang N, Jiang L, Zhu B, Wen Y, Xi XM (2015). Fluid balance and mortality in critically ill patients with acute kidney injury: a multicenter prospective epidemiological study. Crit Care.

[146] Messmer AS, Zingg C, Müller M, Gerber JL, Schefold JC, Pfortmueller CA (2020). Fluid overload and mortality in adult critical care patients—a systematic review and meta-analysis of observational studies*. Crit Care Med.

[147] Aya HD, Ster IC, Fletcher N (2016). Pharmacodynamic analysis of a fluid challenge. Crit Care Med.

[148] Nunes TS, Ladeira RT, Bafi AT (2014). Duration of hemodynamic effects of crystalloids in patients with circulatory shock after initial resuscitation. Ann Intensive Care.

[149] Lankadeva YR, Kosaka J, Iguchi N (2019). Effects of fluid bolus therapy on renal perfusion, oxygenation, and function in early experimental septic kidney injury. Crit Care Med.

[150] Sánchez M, Jimenez-Lendinez M, Cidoncha M (2011). Comparison of fluid compartments and fluid responsiveness in septic and non-septic patients. Anaesth Intensive Care.

[151] Dull RO, Hahn RG (2023). Hypovolemia with peripheral edema: what is wrong?. Crit Care.

[152] Delaney AP, Dan A, McCaffrey J, Finfer S (2011). The role of albumin as a resuscitation fluid for patients with sepsis: a systematic review and meta-analysis. Crit Care Med.

[153] Connolly CM, Kramer GC, Hahn RG, Chaisson NF, Svensén C, Kirschner RA, Hastings DA, Chinkes DL, Prough DS (2003). Isoflurane but not mechanical ventilation promotes extravascular fluid accumulation during crystalloid volume loading. Anesthesiology.

[154] Hahn RG, Lyons G (2016). The half-life of infusion fluids: an educational review. Eur J Anaesthesiol.

[155] Harrois A, Duranteau J (2016). Pathophysiology of severe capillary leak.

[156] Zhang W, Gu Y, Zhao Y (2023). Focused liquid ultrasonography in dropsy protocol for quantitative assessment of subcutaneous edema. Crit Care.

[157] Brodovicz KG, McNaughton K, Uemura N (2009). Reliability and feasibility of methods to quantitatively assess peripheral edema. Clin Med Res.

[158] Lee YH, Lee JD, Kang DR, Hong J, Lee JM (2017). Bioelectrical impedance analysis values as markers to predict severity in critically ill patients. J Crit Care.

[159] Chung YJ, Kim EY (2021). Usefulness of bioelectrical impedance analysis and ECW ratio as a guidance for fluid management in critically ill patients after operation. Sci Rep.

[160] Yilmaz Z (2014). Evaluation of fluid status related parameters in hemodialysis and peritoneal dialysis patients: clinical usefulness of bioimpedance analysis. Medicina (Kaunas).

[161] Buffa R, Mereu E, Comandini O, Ibanez ME, Marini E (2014). Bioelectrical impedance vector analysis (BIVA) for the assessment of two-compartment body composition. Eur J Clin Nutr.

[162] Barbosa-Silva MCG, Barros AJD (2005). Bioelectric impedance and individual characteristics as prognostic factors for postoperative complications. Clin Nutr.

[163] Middel C, Stetzuhn M, Sander N (2023). Perioperative advanced haemodynamic monitoring of patients undergoing multivisceral debulking surgery: an observational pilot study. Intensive Care Med Exp.

[164] Chandra J, Hoz MA, Lee G, Lee A, Thoral P, Elbers P (2022). A novel vascular leak index identifies sepsis patients with a higher risk for in-hospital death and fluid accumulation. Crit Care.

[165] Feldheiser A, Gelman S, Chew M, Stopfkuchen-Evans M (2021). Vasopressor effects on venous return in septic patients: a review. Eur J Anaesthesiol.

[166] Gavelli F, Shi R, Teboul J-L (2022). Extravascular lung water levels are associated with mortality: a systematic review and meta-analysis. Crit Care.

[167] Monnet X, Teboul JL (2017). Transpulmonary thermodilution: advantages and limits. Crit Care.

[168] Tagami T, Kushimoto S, Yamamoto Y, Atsumi T, Tosa R, Matsuda K (2010). Validation of extravascular lung water measurement by single transpulmonary thermodilution: human autopsy study. Crit Care.

[169] Dres M, Teboul JL, Anguel N, Guerin L, Richard C, Monnet X (2014). Extravascular lung water, B-type natriuretic peptide, and blood volume contraction enable diagnosis of weaning-induced pulmonary edema. Crit Care Med.

[170] Kushimoto S, Taira Y, Kitazawa Y (2012). The clinical usefulness of extravascular lung water and pulmonary vascular permeability index to diagnose and characterize pulmonary edema: a prospective multicenter study on the quantitative differential diagnostic definition for acute lung injury/acute respiratory distress syndrome. Crit Care.

[171] Goedhart PT, Khalilzada M, Bezemer R, Merza J, Ince C (2007). Sidestream Dark Field (SDF) imaging: a novel stroboscopic LED ring-based imaging modality for clinical assessment of the microcirculation. Opt Express.

[172] Bruno RR, Hernandez G, Wollborn J (2023). Microcirculation information in clinical decision making: Rome wasn’t built in a day. Intensive Care Med.

[173] Tanaka S, Harrois A, Nicolai C, Flores M, Hamada S, Vicaut E, Duranteau J (2015). Qualitative real-time analysis by nurses of sublingual microcirculation in intensive care unit: the MICRONURSE study. Crit Care.

[174] Naumann DN, Mellis C, Husheer SL, Hopkins P, Bishop J, Midwinter MJ, Hutchings SD (2016). Real-time point of care microcirculatory assessment of shock: design, rationale and application of the point of care microcirculation (POEM) tool. Crit Care.

[175] Bruno RR, Wollborn J, Fengler K (2023). Direct assessment of microcirculation in shock: a randomized-controlled multicenter study. Intensive Care Med.

[176] Lee DH, Dane MJ, Berg BM, Boels MG, Teeffelen JW, Mutsert R, Heijer M, Rosendaal FR, Vlag J, Zonneveld AJ (2014). Deeper penetration of erythrocytes into the endothelial glycocalyx is associated with impaired microvascular perfusion. PLoS ONE.

[177] Ince C, Boerma EC, Cecconi M, Backer D, Shapiro NI, Duranteau J, Pinsky MR, Artigas A, Teboul JL, Reiss IKM (2018). Second consensus on the assessment of sublingual microcirculation in critically ill patients: results from a task force of the European Society of Intensive Care Medicine. Intensive Care Med.

[178] De Backer D, Creteur J, Preiser J-C (2002). Microvascular blood flow is altered in patients with sepsis. Am J Respir Crit Care Med.

[179] Rovas A, Seidel LM, Vink H (2019). Association of sublingual microcirculation parameters and endothelial glycocalyx dimensions in resuscitated sepsis. Crit Care.

[180] Malbrain ML, Mythen MG, Rice TW, Wuyts S (2018). It is time for improved fluid stewardship. ICU Manage Pract.

[181] Ushiyama A, Kataoka H, Iijima T (2016). Glycocalyx and its involvement in clinical pathophysiologies. J Intensive Care.

[182] Alphonsus CS, Rodseth RN (2014). The endothelial glycocalyx: a review of the vascular barrier. Anaesthesia.

[183] Jacob M, Bruegger D, Rehm M (2007). The endothelial glycocalyx affords compatibility of Starling’s principle and high cardiac interstitial albumin levels. Cardiovasc Res.

[184] Adamson RH, Clark JF, Radeva M, Kheirolomoom A, Ferrara KW, Curry FE (2014). Albumin modulates S1P delivery from red blood cells in perfused microvessels: mechanism of the protein effect. Am J Physiol Heart Circ Physiol.

[185] Torres GE, Gainetdinov RR, Caron MG (2003). Plasma membrane monoamine transporters: structure, regulation and function. Nat Rev Neurosci.

[186] Kremer H, Celine B-M, Tesse A, Gallois Y, Mercat A, Henrion D, Andriantsitohaina R, Asfar P, Meziani F (2011). Human serum albumin improves endothelial dysfunction and survival during experimental endotoxemia: concentration-dependent properties. Crit Care Med.

[187] Lang JDJ, Figueroa M, Chumley P (2004). Albumin and hydro- xyethyl starch modulate oxidative inflammatory injury to vascular endothelium. Anesthesiology.

[188] Tatara T (2003). The contribution of solute-solvent exchange at the membrane surface to the reduction by albumin of the hydraulic permeability coefficient of an artificial semipermeable membrane. Anesth Analg.

[189] Cosenza L, Donatti A, Cecero E, Carlucci M, Adembri C, Fusi F (2012). Effects of albumin infusion on LPS-induced damage of mesenteric microcirculation: 12AP1-9. Eur J Anaesthesiol.

[190] Rehm M, Zahler S, Lötsch M, Welsch U, Conzen P, Jacob M, Becker BF (2004). Endothelial glycocalyx as an additional barrier determining extravasation of 6% hydroxyethyl starch or 5% albumin solutions in the coronary vascular bed. Anesthesiology.

[191] Milford EM, Reade MC (2019). Resuscitation fluid choices to preserve the endothelial glycocalyx. Crit Care.

[192] Torres LN, Chung KK, Salgado CL, Dubick MA, Torres Filho IP (2017). Low volume resuscitation with normal saline is associated with microvascular endothelial dysfunction after hemorrhage in rats, compared to colloids and balanced crystalloids. Crit Care.

[193] Hariri G, Joffre J, Maury E (2022). Ten myths about albumin: do not forget the endothelium. Intensive Care Med.

[194] Sakr Y, Bauer M, Nierhaus A (2020). Randomized controlled multicentre study of albumin replacement therapy in septic shock (ARISS): protocol for a randomized controlled trial. Trials.

[195] Raoufinia R, Mota A, Keyhanvar N (2016). Overview of albumin and its purification methods. Adv Pharm Bull.

[196] Zdolsek M, Wuethrich PY, Gunnström M, Zdolsek JH, Hasselgren E, Beilstein CM (2022). Plasma disappearance rate of albumin when infused as a 20% solution. Crit Care.

[197] Zdolsek M, Hahn RG (2022). Kinetics of 5% and 20% albumin: a controlled crossover trial in volunteers. Acta Anaesthesiol Scand.

[198] Hahn RG, Zdolsek M, Hasselgren E, Zdolsek J, Björne H (2019). Fluid volume kinetics of 20% albumin. Br J Clin Pharmacol Juni.

[199] Yanase F, Tosif SH, Churilov L (2021). A randomized, multicenter, open-label, blinded end point, phase 2, feasibility, efficacy, and safety trial of preoperative microvascular protection in patients undergoing major abdominal surgery. Anesth Analg.

[200] Caironi P, Tognoni G, Masson S (2014). Albumin replacement in patients with severe sepsis or septic shock. N Engl J Med.

[201] Pesonen E, Vlasov H, Suojaranta R, Hiippala S, Schramko A, Wilkman E (2022). Effect of 4% albumin solution vs ringer acetate on major adverse events in patients undergoing cardiac surgery with cardiopulmonary bypass: a randomized clinical trial. JAMA.

[202] Lee TH, Kuo G, Chang CH, Huang YT, Yen CL, Lee CC (2021). Diuretic effect of co-administration of furosemide and albumin in comparison to furosemide therapy alone: an updated systematic review and meta-analysis. PLoS ONE.

[203] Wiig H, Swartz MA (2012). Interstitial fluid and lymph formation and transport: physiological regulation and roles in inflammation and cancer. Physiol Rev.

[204] Földi E, Sauerwald A, Hennig B (2000). Effect of complex decongestive physiotherapy on gene expression for the inflammatory response in peripheral lymphedema. Lymphology.

[205] Bozkurt M, Palmer LJ, Guo Y (2017). Effectiveness of decongestive lymphatic therapy in patients with lymphedema resulting from breast cancer treatment regardless of previous lymphedema treatment. Breast J März.

[206] Lasinski BB, McKillip Thrift K, Squire D (2012). A systematic review of the evidence for complete decongestive therapy in the treatment of lymphedema from 2004 to 2011. PM R.

[207] Brix B, Apich G, Roessler A (2020). Fluid shifts induced by physical therapy in lower limb lymphedema patients. JCM.

[208] Schick MA, Wunder C, Wollborn J (2012). Phosphodiesterase-4 inhibition as a therapeutic approach to treat capillary leakage in systemic inflammation. J Physiol.

[209] Wollborn J, Siemering S, Steiger C (2019). Phosphodiesterase-4 inhibition reduces ECLS-induced vascular permeability and improves microcirculation in a rodent model of extracorporeal resuscitation. Am J Physiol Heart Circul Physiol.

[210] Schlegel N, Baumer Y, Drenckhahn D, Waschke J (2009). Lipopolysaccharide-induced endothelial barrier breakdown is cyclic adenosine monophosphate dependent in vivo and in vitro*. Crit Care Medicine.

[211] Spindler V, Schlegel N, Waschke J (2010). Role of GTPases in control of microvascular permeability. Cardiovasc Res.

[212] Schmidt EP, Damarla M, Rentsendorj O (2008). Soluble guanylyl cyclase contributes to ventilator-induced lung injury in mice. Am J Physiol Lung Cell Mol Physiol.

[213] Gonçalves RL, Lugnier C, Keravis T (2009). The flavonoid dioclein is a selective inhibitor of cyclic nucleotide phosphodiesterase type 1 (PDE1) and a cGMP-dependent protein kinase (PKG) vasorelaxant in human vascular tissue. Eur J Pharmacol.

[214] Chan S, Yan C (2011). PDE1 isozymes, key regulators of pathological vascular remodeling. Curr Opin Pharmacol.

[215] Nagel DJ, Aizawa T, Jeon K-I (2006). Role of nuclear Ca^2+^/calmodulin-stimulated phosphodiesterase 1A in vascular smooth muscle cell growth and survival. Circ Res.

[216] Murray F, Patel HH, Suda RY (2007). Expression and activity of cAMP phosphodiesterase isoforms in pulmonary artery smooth muscle cells from patients with pulmonary hypertension: role for PDE1. Am J Physiol Lung Cell Mol Physiol.

[217] Schermuly RT, Pullamsetti SS, Kwapiszewska G (2007). Phosphodiesterase 1 upregulation in pulmonary arterial hypertension target for reverse-remodeling therapy. Circulation.

[218] Vemulapalli S, Watkins RW, Chintala M (1996). Antiplatelet and antiproliferative effects of SCH 51866, a novel type 1 and type 5 phosphodiesterase inhibitor. J Cardiovasc Pharmacol.

[219] Seybold J, Thomas D, Witzenrath M (2005). Tumor necrosis factor a-dependent expression of phosphodiesterase 2: role in endothelial hyperpermeability. Blood.

[220] Dunkern TR, Hatzelmann A (2005). The effect of Sildenafil on human platelet secretory function is controlled by a complex interplay between phosphodiesterases 2, 3 and 5. Cell Signal.

[221] Bubb KJ, Trinder SL, Baliga RS (2014). Inhibition of phosphodiesterase 2 augments cGMP and cAMP signaling to ameliorate pulmonary hypertension. Circulation.

[222] Nikpour M, Sadeghian H, Saberi MR (2010). Design, synthesis and biological evaluation of 6(benzyloxy)-4-methylquinolin-2 (1H)-one derivatives as PDE3 inhibitors. Bioorg Med Chem.

[223] Shakur Y, Holst LS, Landstrom TR (2000). Regulation and function of the cyclic nucleotide phosphodiesterase (PDE3) gene family. Prog Nucleic Acid Res Mol Biol.

[224] Aizawa T, Wei H, Miano JM (2003). Role of phosphodiesterase 3 in NO/cGMP-mediated antiinflammatory effects in vascular smooth muscle cells. Circ Res.

[225] Rotella DP (2002). Phosphodiesterase 5 inhibitors: current status and potential applications. Nat Rev Drug Discov.

[226] Kass DA, Champion HC, Beavo JA (2007). Phosphodiesterase type 5 expanding roles in cardiovascular regulation. Circ Res.

[227] Giannetta E, Feola T, Gianfrilli D (2014). Is chronic inhibition of phosphodiesterase type 5 cardioprotective and safe? A meta-analysis of randomized controlled trials. BMC Med.

[228] Cristina RT, Dehelean C, Dumitrescu E (2010). Structural basis of phosphodiesterase 6 inhibition by the C-terminal region of the g-subunit. EMBO J.

[229] Yang G, McIntyre KW, Townsend RM (2003). Phosphodiesterase 7A-deficient mice have functional T cells. J Immunol.

[230] Redondo M, Brea J, Perez DI (2012). Effect of phosphodiesterase 7 (PDE7) inhibitors in experimental autoimmune encephalomyelitis mice. Discovery of a new chemically diverse family of compounds. J Med Chem.

[231] Wang P, Wu P, Egan RW, Billah MM (2001). Human phosphodiesterase 8A splice variants: cloning, gene organization, and tissue distribution. Gene.

[232] Fisher DA, Smith JF, Pillar JS (1998). Identification and distribution of different mRNA variants produced by differential splicing in the human phosphodiesterase 9A gene. Biochem Biol Chem.

[233] Lee DI, Zhu G, Sasaki T (2015). Phosphodiesterase 9A controls nitric-oxide independent cGMP and hypertrophic heart disease. Nature.

[234] Strick C, Schmidt C, Menniti F (2006). PDE10A: a striatum-enriched, dual-substrate phosphodiesterase, cyclic nucleotide phosphodiesterases in health and disease.

[235] Makhlouf A, Kshirsagar A, Niederberger C (2006). Phosphodiesterase 11: a brief review of structure, expression and function. Int J Impot Res.

[236] Su JB (2015). Vascular endothelial dysfunction and pharmacological treatment. World J Cardiol.

[237] Fulton D, Gratton JP, McCabe TJ (1999). Regulation of endothelium-derived nitric oxide production by the protein kinase Akt. Nature.

[238] Chen CA, Wang TY, Varadharaj S (2010). S-glutathionylation uncouples eNOS and regulates its cellular and vascular function. Nature.

[239] Saravi B, Li Z, Lang CN (2021). The tissue renin–angiotensin system and its role in the pathogenesis of major human diseases: Quo Vadis?. Cells.

[240] Félétou M, Vanhoutte PM (2009). EDHF:an update. Clin Sci.

[241] Fleming I, Michaelis UR, Bredenkötter D (2001). Endothelium-derived hyperpolarizing factor synthase (Cytochrome P450 2C9)is a functionally significant source of reactive oxygen species in coronary arteries. Circ Res.

[242] Blanco-Rivero J, Cachofeiro V, Lahera V (2005). Participation of prostacyclin in endothelial dysfunction induced by aldosterone in normotensive and hypertensive rats. Hypertension.

[243] David S, Russell L, Castro P (2023). Research priorities for therapeutic plasma exchange in critically ill patients. Intensive Care Med Exp.

[244] David S, Bode C, Putensen C (2021). Adjuvant therapeutic plasma exchange in septic shock. Intensive Care Med.

[245] Stahl K, Schmidt JJ, Seeliger B (2020). Effect of therapeutic plasma exchange on endothelial activation and coagulation-related parameters in septic shock. Crit Care.

